# The efficacy and safety of exercise regimens to mitigate chemotherapy cardiotoxicity: a systematic review and meta-analysis of randomized controlled trials

**DOI:** 10.1186/s40959-024-00208-2

**Published:** 2024-02-23

**Authors:** Ahmed Mazen Amin, Yehya Khlidj, Mohamed Abuelazm, Ahmed A. Ibrahim, Mohammad Tanashat, Muhammad Imran, Abubakar Nazir, Hosam Shaikhkhalil, Basel Abdelazeem

**Affiliations:** 1 Faculty of Medicine, Mansoura University, Mansoura, Egypt; 2 Faculty of Medicine, Algiers University, Algiers, Algeria; 3 Faculty of Medicine, Tanta University, Tanta, Egypt; 4 Faculty of Medicine, Menoufia University, Menoufia, Egypt; 5 Faculty of Medicine, Yarmouk University, Irbid, Jordan; 6 University College of Medicine and Dentistry, The University of Lahore, Lahore, Pakistan; 7 Faculty of Medicine, King Edward Medical University, Lahore, Pakistan; 8 Faculty of Medicine, Islamic University of Gaza, Gaza, Palestine; 9 Department of Cardiology, West Virginia University, Morgantown, WV USA

**Keywords:** Exercise, Cancer, Chemotherapy, Cardiotoxic, Review, Meta-analysis

## Abstract

**Background:**

Cardiotoxicity is one of the most common adverse events of the chemotherapy. Physical exercise was shown to be cardioprotective. We aim to estimate the efficacy and safety of exercise in cancer patients receiving cardiotoxic chemotherapy.

**Methods:**

We conducted a systematic review and meta-analysis of randomized controlled trials (RCTs), which were retrieved by systematically searching PubMed, Web of Science, SCOPUS, Cochrane, Clinical Trials.gov, and MedRxiv through July 17th, 2023. We used RevMan V. 5.4 to pool dichotomous data using risk ratio (RR) and continuous data using mean difference (MD), with a 95% confidence interval (CI). PROSPERO ID: CRD42023460902.

**Results:**

We included thirteen RCTs with a total of 952 patients. Exercise significantly increased VO_2_ peak (MD: 1.95 with 95% CI [0.59, 3.32], *P* = 0.005). However, there was no significant effect regarding left ventricular ejection fraction, global longitudinal strain, cardiac output, stroke volume, left ventricular end-diastolic volume, left ventricular end-systolic volume, E/A ratio, resting heart rate, peak heart rate, resting systolic blood pressure, and resting diastolic blood pressure. Also, there was no significant difference regarding any adverse events (AEs) (RR: 4.44 with 95% CI [0.47, 41.56], *P* = 0.19), AEs leading to withdrawal (RR: 2.87 with 95% CI [0.79, 10.43], *P* = 0.11), serious AEs (RR: 3.00 with 95% CI [0.14, 65.90], *P* = 0.49), or all-cause mortality (RR: 0.25 with 95% CI [0.03, 2.22], *P* = 0.21).

**Conclusion:**

Exercise is associated with increased VO_2_ peak in cancer patients receiving cardiotoxic chemotherapy. However, there was no significant difference between exercise and usual care regarding the echocardiographic and safety outcomes.

**Supplementary Information:**

The online version contains supplementary material available at 10.1186/s40959-024-00208-2.

## Introduction

Chemotherapy-induced cardiotoxicity (CIC) refers to the direct and indirect adverse effects of different chemotherapeutic agents on the cardiovascular system [1]. In particular, the incidence of left ventricular dysfunction among patients treated with certain anticancer drugs, such as doxorubicin at high doses (700 mg/m^2^), can reach 48%. In contrast, the incidence of myocardial ischemia due to 5-fluorouracil (5-FU) is reported to be as high as 10% [2, 3]. Moreover, 26–93% of patients on arsenic trioxide show prolonged QT interval, and many develop life-threatening ventricular tachyarrhythmias [4]. Besides being a not infrequently occurring event, CIC corresponds to a wide range of adverse events. According to the European Society of Cardiology’s Task Force for Cancer Treatments and Cardiovascular Toxicity, chemotherapy-related cardiovascular complications are classified as myocardial dysfunction and heart failure, coronary artery disease (CAD), arrhythmias, arterial hypertension, thromboembolic disease, peripheral vascular disease, pulmonary hypertension, and pericardial complications [2].

Consequently, different pharmacological and non-pharmacological therapies were investigated as potential preventive approaches against CIC, among them physical exercise, whose efficacy and tolerability were tested by numerous clinical trials with promising results [5, 6]. Several parameters can be used to assess the effects of exercise on cardiac function, such as left ventricular ejection fraction (LVEF), left ventricular end-diastolic volume (LVEDV), left ventricular end-systolic volume (LVESV), and global longitudinal strain (GLS) which are all echocardiographically determined [7]. Besides this, cardiovascular fitness, i.e., peak oxygen uptake (VO_2_ peak) is also an interesting outcome to evaluate in this context. VO_2_ peak our primary outcome, is the peak value of oxygen uptake attained during exercise [8]. In a recent meta-analysis, high-intensity interval training positively affected cancer patients' functional performance [6]. Similarly, it was reported that exercise training can ameliorate cardiorespiratory fitness following chemotherapy with anthracyclines [9]. Additionally, the randomized controlled trial (RCT) known as The BREXIT Study has demonstrated that exercise can effectively prevent anthracycline-induced functional disability and cardiac impairment [10]. In contrast, another RCT has concluded the lack of feasibility of intensive aerobic training in a significant proportion of patients with metastatic breast cancer receiving chemotherapy [11].

Thus, it is not clear if the current data is sufficient to encourage the use of exercise for patients at risk of CIC, especially since exercise is not currently a part of the recommended standards of care for cancer management [12]. Furthermore, most established cardio-protective exercise abilities were observed in non-cancer populations [5]; therefore, the same effects may not necessarily be seen in cancer survivors.

This creates a solid rationale to extensively examine the findings of the current literature to provide a vigorous assessment of exercise advantages in lowering the risks of cardiovascular events following chemotherapy. Consequently, in the present systematic review and meta-analysis, we explored the quality of evidence that determines exercise's cardiac efficacy and safety in patients receiving chemotherapy. Our work may lead to insightful findings that can have key therapeutic implications.

## Methodology

### Protocol registration

The PRISMA statement and the Cochrane Handbook for systematic reviews and meta-analyses were followed to conduct this systematic review and meta-analysis [13, 14]. This meta-analysis process has been registered and published in PROSPERO under the following ID: CRD42023460902.

### Data sources & search strategy

PubMed (MEDLINE), Scopus, Cochrane Central Register of Controlled Trials (CENTRAL), Web of Science Core Collection, EMBASE, Clinical Trials.gov, and MedRxiv were systematically searched until July 17th, 2023. We modified search terms and keywords for each database, as presented in (Table S1).

### Eligibility criteria

We included randomized controlled trials (RCTs) published in English language that followed the following PICO criteria: population (P): patients diagnosed with any type of cancer receiving any cardiotoxic chemotherapeutic agent; intervention (I): any form of supervised aerobic or resistance exercise training irrespective of the exercise duration, frequency and intensity; control (C): usual care without any form of exercise training; and outcomes (O): primary outcome of this review is the VO_2_ peak. While our secondary outcomes include left ventricular ejection fraction (LVEF) change, change in global longitudinal strain (GLS), cardiac output (CO) (L/min) change, stroke volume (SV) (ml) change, left ventricular end-diastolic volume (LVEDV) (ml) change, left ventricular end-systolic volume (LVESV) (ml) change, E/A ratio change, respiratory exchange ratio (RER) change, resting heart rate (RHR) change, peak heart rate (PHR) change, resting systolic blood pressure (RSBP) (mmHg) change, resting diastolic blood pressure (RDBP) (mmHg) change, and safety outcomes, including the incidence of any adverse events, any serious adverse events, any adverse events leading to withdrawal, and mortality.

### Study selection

To perform the review, we used the Covidence web tool. After deleting duplicates, four authors (M.T., M.I., A.N., and H.S.) independently evaluated the obtained records. Four authors (M.T., M.I., A.N., and H.S.) checked the full texts of the records that satisfied the initial eligibility criterion during the full-text screening. Any differences were settled by discussion and agreement with B.A.

### Data extraction

We conducted a pilot extraction after retrieving the complete texts of relevant papers in order to prepare the data extraction sheet appropriately. The data extraction sheet, which is structured in Excel (Microsoft, USA), is divided into three sections. The first part included the summary characteristics of the included studies (name of first author, year of publication, country, exercise intensity, intervention frequency (Sessions per week), chemotherapeutic drug, exercise adherence, cancer type, cancer stage, and study design). The second part included the baseline information of the participants (sample size, age, menopausal status, body mass index (BMI), cancer stage, and comorbidities). Finally, the third part included outcomes data as previously described. Four reviewers (M.T., M.I., A.N., and H.S.) were responsible for data extraction. Any differences were settled by discussion and agreement with B.A.

### Risk of bias and certainty of evidence

Using the Cochrane RoB2 tool, four reviewers (M.T., M.I., A.N., and H.S.) independently evaluated the quality of the studies [15]. They assessed five domains, including the risk of bias associated with the randomization process, deviation from the intended intervention, missing outcome data, measuring the outcome, and choosing the reported results. Any differences were settled by discussion and agreement with B.A. Two reviewers (M.A. and B.A.) followed the Grading of Recommendations Assessment, Development, and Evaluation (GRADE) criteria [16, 17] to evaluate the certainty of evidence. Any disagreements were resolved through consensus.

### Statistical analysis

The RevMan v5.3 software was used for the statistical analysis [18]. We employed the risk ratio (RR) to combine the results of dichotomous outcomes and the mean difference (MD) for continuous outcomes, both with a 95% confidence interval (CI), using the fixed-effects model. However, the random-effects model was used in case of significant heterogeneity. To assess heterogeneity, we utilized the Chi-square and I-square tests, where the Chi-square test establishes if heterogeneity exists, and the I-square test assesses the level of heterogeneity. According to the Cochrane Handbook (chapter nine) [19], we considered an alpha level of less than 0.1 for the Chi-square test to indicate significant heterogeneity, while an I-square more than 75% indicated considerable heterogeneity. When there was significant heterogeneity, sensitivity analysis was used in which we excluded one study in each scenario to detect possible heterogeneity causes.

Trial Sequential Analysis (TSA) was employed to assess the conclusiveness and reliability of the data of the pooled trials and to assess if the sample size of the current meta-analysis was adequate to make solid conclusions regarding the impact of the interventions. When the Z-line on the curve cut both the conventional and trial sequential monitoring boundary (TSMB), we assumed that the intervention's confidence level was conclusive and sufficient and that no additional studies were required. However, if the Z-line does not cut any boundaries, the evidence is insufficient, and further studies are needed [20, 21]. In this meta-analysis, we utilized an alpha error of 0.05, a beta error of 80% power, and a predicted RR reduction of 20% in dichotomous outcomes. Moreover, we made a subgroup analysis based on exercise type (aerobic exercise, restrictive exercise, and combined aerobic and restrictive exercise) and regarding whether the patients had breast cancer only or breast cancer plus other cancers throughout our primary and echocardiographic outcomes to detect possible differences between the subgroups.

## Results

### Search results and study selection

**Fig. 1:**
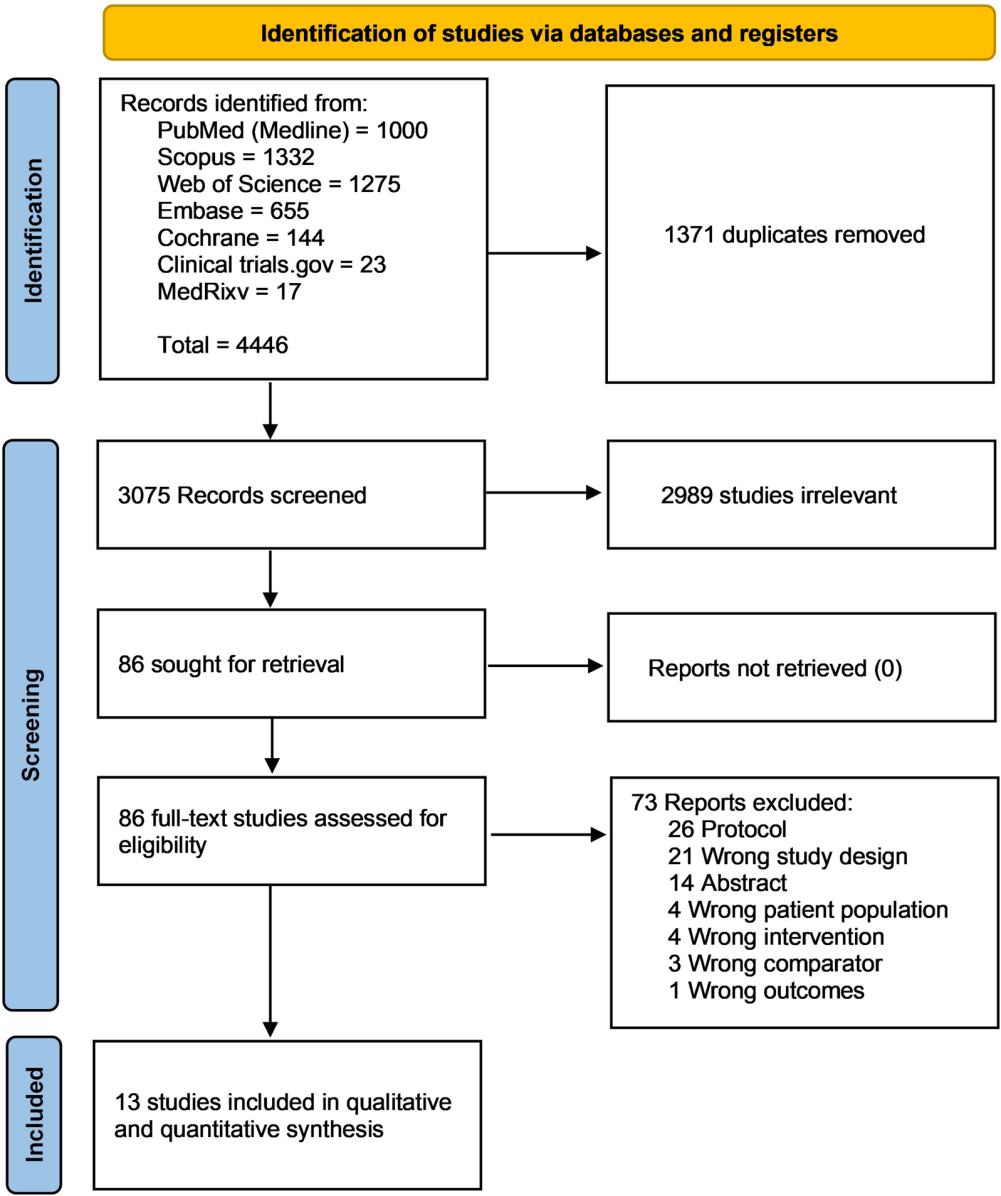
PRISMA flow chart of the screening process

This literature search from PubMed (MEDLINE), Scopus, Cochrane Central Register of Controlled Trials (CENTRAL), Web of Science Core Collection, EMBASE, Clinical Trials.gov, and MedRxiv yielded a total of 4,446 articles. After duplication removal (n = 1371) and reviewing the title and abstract (n = 3075) for relevance, eighty-six articles were left for full-text screening. Thirteen of these studies met the inclusion criteria for our systematic review and meta-analysis. The PRISMA flow diagram displays the search results and studies selection process (Fig. 1).

### Characteristics of included studies

This study involves thirteen RCTs [9, 10, 22–32] with a total of 952 patients, diagnosed with various types of cancer undergoing treatment with cardiotoxic chemotherapeutic agents. Among them, 569 (59.77%) patients participated in supervised aerobic or resistance exercise training sessions, whereas 383 (40.23%) did not receive any type of exercise. All the RCTs included the participants with breast cancer except Tsai et al. 2019 [24], which included the patients with Sarcoma hip/thigh, Lymphoma, Multiple myeloma, Osteosarcoma, Hodgkin's disease, and Leukemias as well. Also, in all the included RCTs, participants were delivered moderate to vigorous intensity exercise; however, there were variations in the exercise character, duration, and the number of exercise sessions among the studies. The detailed summary characteristics of the included RCTs and participants’ baseline characteristics are shown in (Table 1 and 2) respectively.

**Table 1:** Characteristics of the included studies

Study ID	Study Design	Country	Total Participants	Intervention						Chemotherapeutic drug	Cancer Type and Stage	Exercise Adherence	Primary Outcome	Follow-up duration
				**Intervention**	**Intervention duration (Weeks)**	**Intervention frequency (Sessions per week)**	**Session duration (min)**	**Exercise intensity**	**Control**					
**Bolam et al.2019** [25]** (OptiTrain)**	RCT	Sweden	260	1) Resistance and high-intensity interval training (RT-HIIT) OR 2) Moderate-intensity aerobic and high-intensity interval training (AT–HIIT)	16	2	60	Moderate-to-high–intensity exercise	Usual care	Anthracyclines, taxanes, or a combination of the two	Breast cancer stage I–IIIa	NR	primary outcome fatigue measured by the Piper Fatigue scale	2 years
**Antunes et al. 2023** [9]	Single center Randomized Controlled trial	Portugal	93	Combining aerobic and resistance training	20—24	3	35—55	Moderate and vigorous intensity	Usual care	Doxorubicin plus cyclophosphamide +—taxane-based chemotherapy +—plus trastuzumab with or without pertuzumab +—carboplatin and paclitaxel	Breast cancer stage I-III	Mean Adherence 63.2 ± 26.9%	The absolute change in ventricle ejection fraction (LVEF) from baseline to the end of anthracycline cycles	[20–24 weeks intervention] + 3 months follow up
**Chung et al. 2022** [26]	Open-labelled single center Randomized Controlled trial	Taiwan	32	Real-time exercise (aerobic exercise, resistance exercise, and flexibility training)	12	2 to 3	65	Moderate-to-high–intensity exercise	Usual care	Chemotherapy every 3 weeks with CEF for 6 cycles (cyclophosphamide 500 mg/m2, epirubicin 75 mg/m2, and 5-FU 500 mg/m2) or doxorubicin plus cyclophosphamide for 4 cycles followed by docetaxel for 4 cycles (doxorubicin 60 mg/m2, cyclophosphamide 600 mg/m2 and docetaxel 60 mg/m2	Breast cancer stage I–III	76%	The change in left LVEF	12 months
**Foulkes et al. 2023** [10]**(The BREXIT)**	Open-labelled single center Randomized Controlled trial	Australia	104	Aerobic and resistance Exercise training	52	3 to 4	80 -120	Moderate-to-high–intensity exercise	Usual care	Anthracycline-based chemotherapy +—Taxane +—carboplatin chemotherapy + -Capecitabine chemotherapy	Breast cancer stage I-III	73%	Functional disability at 12 months, defined as a VO_2_ peak ≤ 18.0 mL∙kg − 1∙min − 1	12 months
**Hojan et al. 2020** [28]** (REH-HER)**	Open-labelled single center Randomized Controlled trial	Poland	68	Regular aerobic/resistance exercise	9	5	85—95	Moderate intensity exercise	General physical activity	Trastuzumab	Breast cancer stage I–IIIa	98.7%	The differences in cardiac function measured with a capacity test over the nine weeks of the exercise program	9 weeks
**Hornsby et al. 2014** [29]	RCT	USA	20	Aerobic training consisted of one-on-one (nongroup based) supervised cycle ergometry sessions	12	3	15–45	Moderate- to high-intensity exercise	Usual care	Doxorubicin & Cyclophosphamide	Stage IIB–IIIC breast adenocarcinoma	66%	Safety outcomes included exercise testing as well as treatment- and exercise training-related adverse events (AEs), whereas efficacy outcomes included cardiopulmonary function and patient-reported outcomes (PROs) as measured by a cardiopulmonary exercise test (CPET) and Functional Assessment of Cancer Therapy-Breast (FACT-B) scale	12 weeks
**Jacquinot et al. 2022** [30]	Multicenter Randomized Controlled trial	France	89	Supervised exercise program (aerobic)	12	3	55	Moderate- to high-intensity exercise	Usual care	Trastuzumab	Breast cancer	NR	Test whether trastuzumab induced cardiotoxicity [left ventricular ejection fraction (LVEF) under 50%, or an absolute drop in LVEF of 10%] was reduced after a supervised exercise program of 3 months in patients with HER2-positive breast cancer	6 months
**Kerrigan et al. 2023** [31]	Multicenter Randomized Controlled trial	USA	29	interval training protocol with 4-min high-intensity intervals alternated by 3 min of moderate intensity. (aerobic)	10	2 to 3	40—50	moderate- to high-intensity exercise	Usual care	doxorubicin and/or trastuzumab	breast cancer stages I-IV and leiomyosarcoma	59%	Our primary aim was to determine whether CR improves exercise capacity in patients who have exhibited subclinical markers of myocardial damage due to doxorubicin or trastuzumab	10 weeks
**Kirkham et al. 2018** [32]	Randomized Controlled trial	Canada	27	Supervised treadmill exercise (aerobic)	4 Sessions performed, each 24 h prior to each episode of treatment. Up to 2 weeks	NR	10-min warm-up, 30 min of vigorous and a 5-min cool-down	moderate-to-vigorous physical activity	Abstain from vigorous-intensity exercise from 72 h prior to, and 48 h after the treatment	Doxorubicin	Breast Cancer, stage I–III	94% adherence to timing, 83% adherence to intensity,98% adherence to duration	To investigate the effect of this intervention on established markers of subclinical cardiotoxicity at the end of treatment	7–14 days
**Lee et al. 2019** [22]	Randomized pilot clinical trial	USA	30	High intensity interval training (aerobic)	8	3 times	30	Walking + Moderate + Vigorous	non-exercise	Doxorubicin & cyclophosphamide	Breast Cancer, stage I–III	82.3% in HIT group	VO_2_ max change	9 weeks
**Sturgeon et al. 2022** [23]	Randomized controlled trial	USA	19	Tailored home-based remotely delivered (aerobic exercise)	24 weeks	From week 1–4, 3 sessions/wk with a total of 60min/week at 50% of baseline VO_2_ max and to 75 + min/wk at 60% of VO_2_ max at the end of week 4, From week 5–24, 2 sessions/ week at 65–75% of baseline VO_2_ max	N/R	moderate-to-vigorous	usual level of physical activity	Neoadjuvant with Taxotere, Carboplatin, Herceptin + Perjeta; TCH + P, OR, Adriamycin, cyclophosphamide, Taxol; ACT	Breast Cancer, stage I–III	87.60%	VO_2_ max change	16–24 weeks
**Tsai et al. 2019** [24]	Randomized controlled trial	USA	22	Clinic and home-based exercise intervention (aerobic exercise)	16 weeks	3 times	30 min	moderate-to-vigorous	non-exercising	Non-specific	Breast, Sarcoma hip/thigh, Lymphoma, Multiple myeloma, Osteosarcoma, Hodgkin's disease, Leukemia	NR	VO_2_ max change	16 weeks
**Courneya et al. 2007** [27]	Multicenter prospective, three-armed, randomized controlled trial	Canada	242	Aerobic exercise training (AET) and resistance exercise training (RET)	Median 17 weeks, 95% CI (9 to 24 weeks)	3 times	15 min for weeks 1 to 3, increased by 5 min every 3weeks till 45 min at week 18	Vigorous	Usual care	Nontaxane and Taxane both	Breast cancer stage, I–IIIA	70.2%	Cancer-specific QOL assessed by the Functional Assessment of Cancer Therapy–Anemia scale	(9–24 chemotherapy treatment) + 3 to 4 weeks after chemotherapy

*NR* Not Reported

**Table 2:** Baseline characteristics of the participants included

Study ID	Number of patients in each group		Age (Years), Mean (SD)		BMI, Mean (SD)		Menopausal status N. (%)				Cancer stage N. (%)				
	**Intervention**	**Control**	**Intervention**	**Control**	**Intervention**	**Control**	**Premenopausal**		**Postmenopausal**		**1**		**2**		**3**
							**Intervention**	**control**	**Intervention**	**control**	**Intervention**	**control**	**Intervention**	**control**	**Intervention**
**Bolam et al.2019** [25]**(OptiTrain) (RET group)**	74	60	52.7 (10.3)	52.6 (10.2)	25.1 (4.3)	24.6 (4.8)	36 (48.6)	23 (38.3)	38 (51.4)	37 (61.7)	NR	NR	NR	NR	NR
**Bolam et al.2019** [25]**(OptiTrain) (AET group)**	72	60	54.4 (10.3)	52.6 (10.2)	24.8 (4.4)	24.6 (4.8)	26 (36.1)	23 (38.3)	46 (63.9)	37 (61.7)	NR	NR	NR	NR	NR
**Antunes et al. 2023** [9]	47	46	49.66 (9.43)	51.02 (9.54)	26.94 (4.32)	28.69 (6.82)	29 (61.7)	24 (52.2)	18 (38.3)	22 (47.8)	7 (14.9)	7 (15.2)	26 (55.3)	21 (45.7)	14 (29.8)
**Chung et al. 2022** [26]	16	13	52.4 (8.9)	50.3 (7.7)	24.6 (6.1)	23.2 (2.7)	6 (30)	5 (38)	10 (70)	8 (62)	7 (43.75)	7 (54)	5 (31.25)	6 (46)	4 (25)
**Foulkes et al. 2023** [10]** (The BREXIT)**	52	50	50.3 (7.7)	51.2 (7.6)	27.5 (4.6)	27.5 (5.6)	31 (60)	25 (50)	21 (40)	25 (50)	4 (8)	1 (2)	25 (48)	34 (68)	23 (44)
**Hojan et al. 2020** [28]** (REH-HER)**	26	21	54.44 (6.29)	54.64 (5.26)	24.35 (2.8)	25.35 (1.89)	NR	NR	NR	NR	2 (7.7%)	0 (0)	21 (80.7)	21 (100)	3 (11.5%)
**Hornsby et al. 2014** [29]	10	10	51(6)	46(11)	29(5)	28(9)	NR	NR	NR	NR	NR	NR	NR	NR	NR
**Jacquinot et al. 2022** [30]	46	43	51.1 (4.87)	51.0 (10.39)	24.7 (8.271)	26.0 (2.741)	NR	NR	NR	NR	3(6.7)	1(2.4)	17(37.8)	15(35.7)	25(55.6)
**Kerrigan et al. 2023** [31]	11	11	58 (11)	52 (13)	31 (7)	34 (5)	NR	NR	NR	NR	0 (0)	3 (27)	8 (72)	5 (45)	2 (18)
**Kirkham et al. 2018** [32]	13	11	52 (9)	51 (10)	25.0 (4.8)	26.7 (5.1)	4 (31)	4 (36)	4 (31)	6 (55)	1 (8)	3 (27)	7 (54)	5 (45)	5 (38)
**Lee et al. 2019** [22]	15	15	49.1 (7.9)	44.7 (11.2)	33.1 (7.6)	30.1 (7.7)	5 (33)	6 (40)	10 (67)	9 (60)	1 (6)	1 (6)	5 (30)	4 (24)	9 (64)
**Sturgeon et al. 2022** [23]	9	10	47.0 (11.7)	51.5 (9.5)	NR	NR	NR	NR	NR	NR	2 (22)	2 (20)	5 (55)	5 (50)	2 (22)
**Tsai et al. 2019**(24)	14	8	54 (10.02)	55.2 (13.5)	30.89 (9.06)	30.2 (5.7)	NR	NR	NR	NR	NR	NR	NR	NR	NR
**Courneya et al. 2007 (RET group)** [27]	82	82	49.5	49	26.1 (5.5)	27.1 (5.4)	47 (57.3)	55 (67)	35 (42.7)	27(32.9)	22 (26.8)	20 (24.4)	45 (55)	52 (63)	15 (18.3)
**Courneya et al. 2007 (AET group)** [27]	78	82	49	49	26.7 (5.6)	27.1 (5.4)	51 (65.4)	55(82)	27 (34.6)	27(32.9)	18 (23.1)	20 (24.4)	50 (64.1)	52 (63)	10 (12.8)

Study ID				Comorbidities N. (%)								Chemotherapy N. (%)			
	**3**	**4**		**Smoker**		**Diabetes**		**Obesity**		**Hyperlipidemia**		**Neoadjuvant**		**Adjuvant**	
	**control**	**Intervention**	**control**	**Intervention**	**control**	**Intervention**	**control**	**Intervention**	**control**	**Intervention**	**control**	**Intervention**	**control**	**Intervention**	**control**
**Bolam et al.2019** [25]**(OptiTrain) (RET group)**	NR	NR	NR	3 (4.3)	3 (5.2)	NR	NR	NR	NR	NR	NR	0 (0)	0 (0)	74 (100)	60 (100)
**Bolam et al.2019** [25]**(OptiTrain) (AET group)**	NR	NR	NR	4 (5.9)	3 (5.2)	NR	NR	NR	NR	NR	NR	0 (0)	0 (0)	72 (100)	60 (100)
**Antunes et al. 2023** [9]	14 (29.8)	0	0	10 (21.3)	8 (17.4)	2 (4.3)	5 (10.9)	12 (25.5)	15 (32.6)	NR	NR	30 (63.8)	34 (73.9)	17 (36.9)	12 (26.1)
**Chung et al. 2022** [26]	0	0	0	NR	NR	1 (6.25)	2 (15.38)	NR	NR	0 (0)	3 (23.07)	1 (6)	0	15 (94)	13 (100)
**Foulkes et al. 2023** [10]** (The BREXIT)**	15 (30)	0	0	NR	NR	1 (2)	1 (2)	38 (73)	30 (60)	2 (4)	0 (0)	35 (67)	32 (64)	17 (33)	18 (36)
**Hojan et al. 2020** [28]** (REH-HER)**	0 (0)	0 (0)	0 (0)	6 (23%)	2 (9.5%)	1 (3.8%)	0 (0)	NR	NR	4 (15.4%)	2 (9.5%)	NR	NR	NR	NR
**Hornsby et al. 2014** [29]	NR	NR	NR	NR	NR	NR	NR	NR	NR	NR	NR	10 (100)	10 (100)	0 (0)	0 (0)
**Jacquinot et al. 2022** [30]	26(61.9)	NR	NR	11 (23.9)	5 (11.6)	NR	NR	NR	NR	NR	NR	0 (0)	0 (0)	46 (100)	43 (100)
**Kerrigan et al. 2023** [31]	2 (18)	1 (9)	1 (9)	NR	NR	NR	NR	NR	NR	NR	NR	NR	NR	NR	NR
**Kirkham et al. 2018** [32]	3 (27)	0	0	0	0	0 (0)	1 (9)	NR	NR	0	0	4 (31)	4 (36)	9 (69)	7 (63)
**Lee et al. 2019** [22]	10 (70)	0	0	0	0	NR	NR	NR	NR	NR	NR	11 (73)	12 (80)	4 (27)	3 (20)
**Sturgeon et al. 2022** [23]	3 (30)	0	0	2 (22)	1 (10)	NR	NR	NR	NR	NR	NR	9 (100)	10 (100)	0 (0)	0 (0)
**Tsai et al. 2019**(24)	NR	NR	NR	NR	NR	NR	NR	NR	NR	NR	NR	0 (0)	0 (0)	14 (100)	8 (100)
**Courneya et al. 2007 (RET group)** [27]	10 (12)	0 (0)	0 (0)	9 (11)	5 (6)	NR	NR	14 (17.1)	19 (23)	NR	NR	0	0	82	82
**Courneya et al. 2007 (AET group)** [27]	10 (12)	0 (0)	0 (0)	6 (7.7)	5 (6)	NR	NR	17 (21.8)	19 (23)	NR	NR	0	0	78	82

*BMI* Body Mass Index, *SD* Standard Deviation, *N. (%)*Number (Percentage), *RET* Resistant Exercise Training, *AET* Aerobic Exercise Training, *NR* Not Reported

### Risk of bias and certainty of evidence

**Fig. 2:**
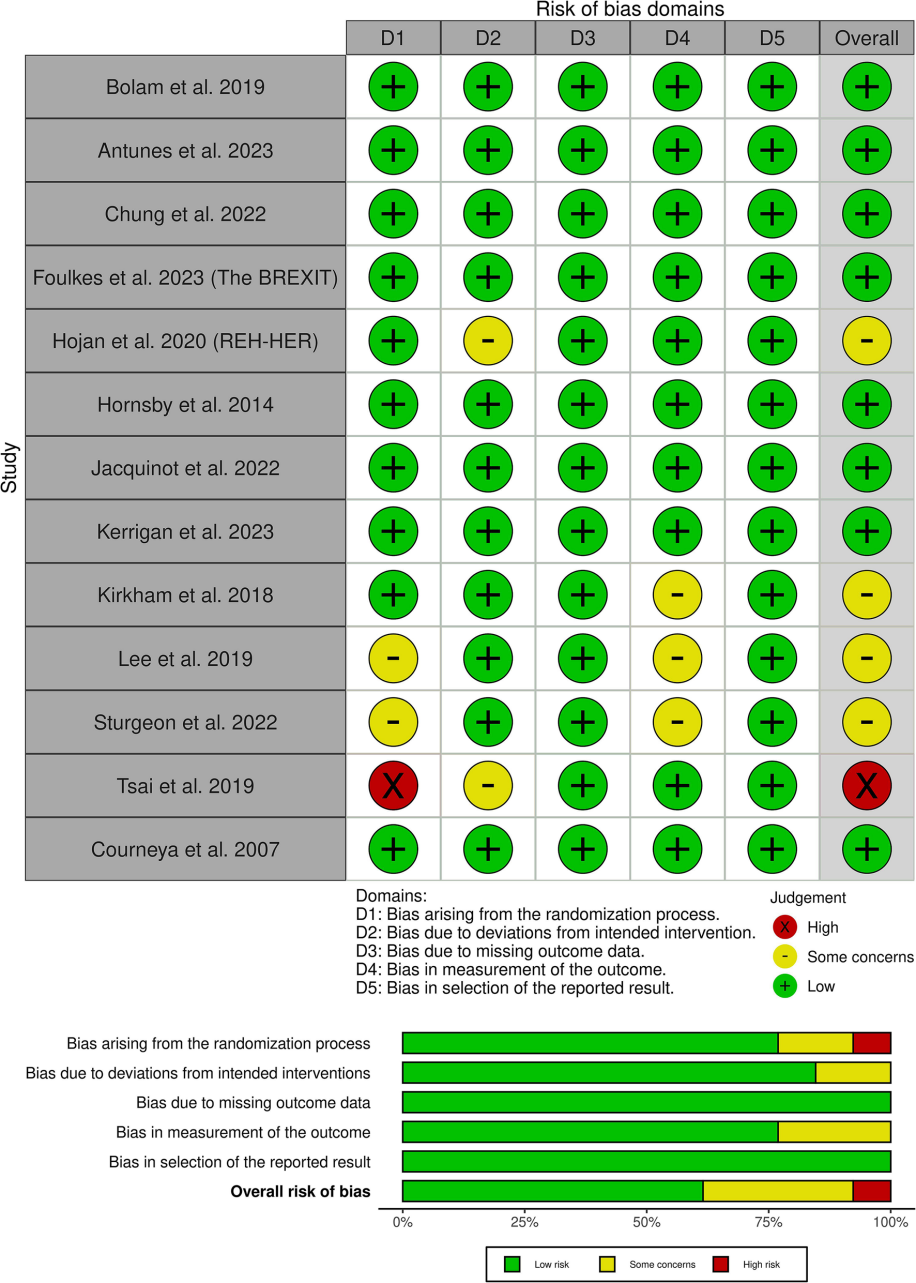
Quality assessment of the risk of bias in the included trials. The upper panel presents a schematic representation of risks (low = green, unclear = yellow, and high = red) for specific types of biases of each of the studies in the review. The lower panel presents risks (low = green, unclear = yellow, and high = red) for the subtypes of biases of the combination of studies included in this review

The risk of bias assessment for each outcome is presented in (Fig. 2). Overall, most of the included studies demonstrated a low risk of bias across all assessed domains. Specifically, four studies raised some concerns regarding the risk of bias, primarily stemming from issues related to outcome measurement. Notably, only one study was deemed to have a high risk of bias, primarily due to shortcomings in the randomization process. More details about the authors’ decision are in (Table S2). Certainty of evidence is demonstrated in a GRADE evidence profile (Table 3).

**Table 3:** GRADE evidence profile

Certainty assessment						
**Participants (studies)** **Follow-up**	**Risk of bias**	**Inconsistency**	**Indirectness**	**Imprecision**	**Publication bias**	**Overall certainty of evidence**
**VO**_**2**_** peak, ml/kg/min Change**						
777 (8 RCTs)	not serious	very serious^a^	not serious	not serious	none	⨁⨁◯◯ Low
**Echocardiographic outcomes—Left Ventricular Ejection fraction (%) change**						
403 (8 RCTs)	not serious	not serious	not serious	very serious^b^	none	⨁⨁◯◯ Low
**Echocardiographic outcomes—Global Longitudinal strain (%) Change**						
332 (6 RCTs)	not serious	serious^c^	not serious	very serious^b^	none	⨁◯◯◯ Very low
**Echocardiographic outcomes—Stroke volume (ml) change**						
260 (5 RCTs)	not serious	very serious^a^	not serious	extremely serious^b^	none	⨁◯◯◯ Very low
**Echocardiographic outcomes—Left Ventricular end-diastolic volume (ml) change**						
166 (4 RCTs)	not serious	not serious	not serious	extremely serious^b^	none	⨁◯◯◯ Very low
**Echocardiographic outcomes—Left Ventricular end-systolic volume (ml) change**						
166 (4 RCTs)	not serious	not serious	not serious	very serious^b^	none	⨁⨁◯◯ Low
**Echocardiographic outcomes—E/A ratio change**						
295 (5 RCTs)	not serious	not serious	not serious	serious^d^	none	⨁⨁⨁◯ Moderate
**Echocardiographic outcomes—Cardiac output (L/min) change**						
239 (4 RCTs)	not serious	very serious^a^	not serious	serious^b^	none	⨁◯◯◯ Very low
**Adverse events—Any adverse event**						
227 (6 RCTs)	not serious	serious^c^	not serious	very serious^e^	none	⨁◯◯◯ Very low
**Adverse events—Any serious advere event**						
249 (7 RCTs)	not serious	not serious	not serious	very serious^e^	none	⨁⨁◯◯ Low
**Adverse events—Any advere event leading to withdrawal**						
295 (7 RCTs)	not serious	not serious	not serious	very serious^e^	none	⨁⨁◯◯ Low
**Adverse events—All-Cause Mortality**						
295 (7 RCTs)	not serious	not serious	not serious	very serious^e^	none	⨁⨁◯◯ Low
**RER Change**						
173 (4 RCTs)	not serious	not serious	not serious	very serious^f^	none	⨁⨁◯◯ Low
**Resting Heart rate (BPM) Change**						
215 (5 RCTs)	not serious	not serious	not serious	very serious^b^	none	⨁⨁◯◯ Low
**Peak Heart rate (BPM) Change**						
258 (6 RCTs)	not serious	not serious	not serious	very serious^b^	none	⨁⨁◯◯ Low
**Resting Systolic blood pressure (mmHg) Change**						
113 (4 RCTs)	not serious	not serious	not serious	very serious^b^	none	⨁⨁◯◯ Low
**Resting Diastolic blood pressure (mmHg) Change**						
113 (4 RCTs)	not serious	not serious	not serious	very serious^b^	none	⨁⨁◯◯ Low

*CI* confidence interval, *MD* mean difference, *RR* risk ratio

**Explanations**

^a^I-square > 75%

^b^Wide confidence interval and number of patients is less than 400 patient

^c^I-square > 50%

^d^Number of patients is less than 400 patients

^e^Wide confidence interval that does not exclude the appreciable benefit or harm

^f^Number of events is less than 300 event

### Primary outcome

**Fig. 3:**
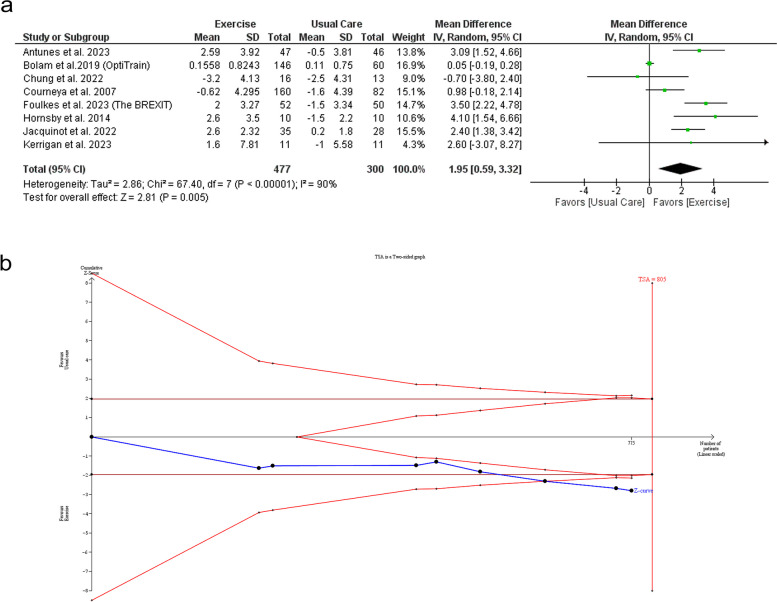
Forest plot and trial sequential analysis of the primary efficacy outcome (VO_2_ peak), MD: mean difference, CI: confidence interval

There was a significant difference between exercise and usual care regarding VO_2_ peak change with (MD: 1.95 with 95% CI [0.59 -3.32], *P* = 0.005) (Fig. 3-A). The pooled studies were heterogeneous (I^2^ = 90%, *P* < 0.00001). Heterogeneity was not resolved by leave-one-out sensitivity analysis (Table S3). TSA showed that the available evidence crossed both the conventional boundary and TSMB, indicating robust conclusions (Fig. 3-B). The subgroup analysis showed a significant difference in exercise type subgroups (*P* = 0.006) with a significant increase in VO_2_ peak in the aerobic exercise group (MD: 1.89 with 95% CI [0.23 – 3.55], *P* = 0.03), and combined exercise group (MD: 2.47 with 95% CI [0.63 – 4.30], *P* = 0.008). However, there was no difference in the resistant exercise group (MD: 0.10 with 95% CI [-0.16 – 0.37], *P* = 0.44) (Figure S1). However, test for subgroup analysis was not significant regarding whether the patients had breast cancer only or breast cancer plus other cancers (*P* = 0.82) (Figure S2).

### Secondary outcomes

#### Efficacy outcomes

**Fig. 4:**
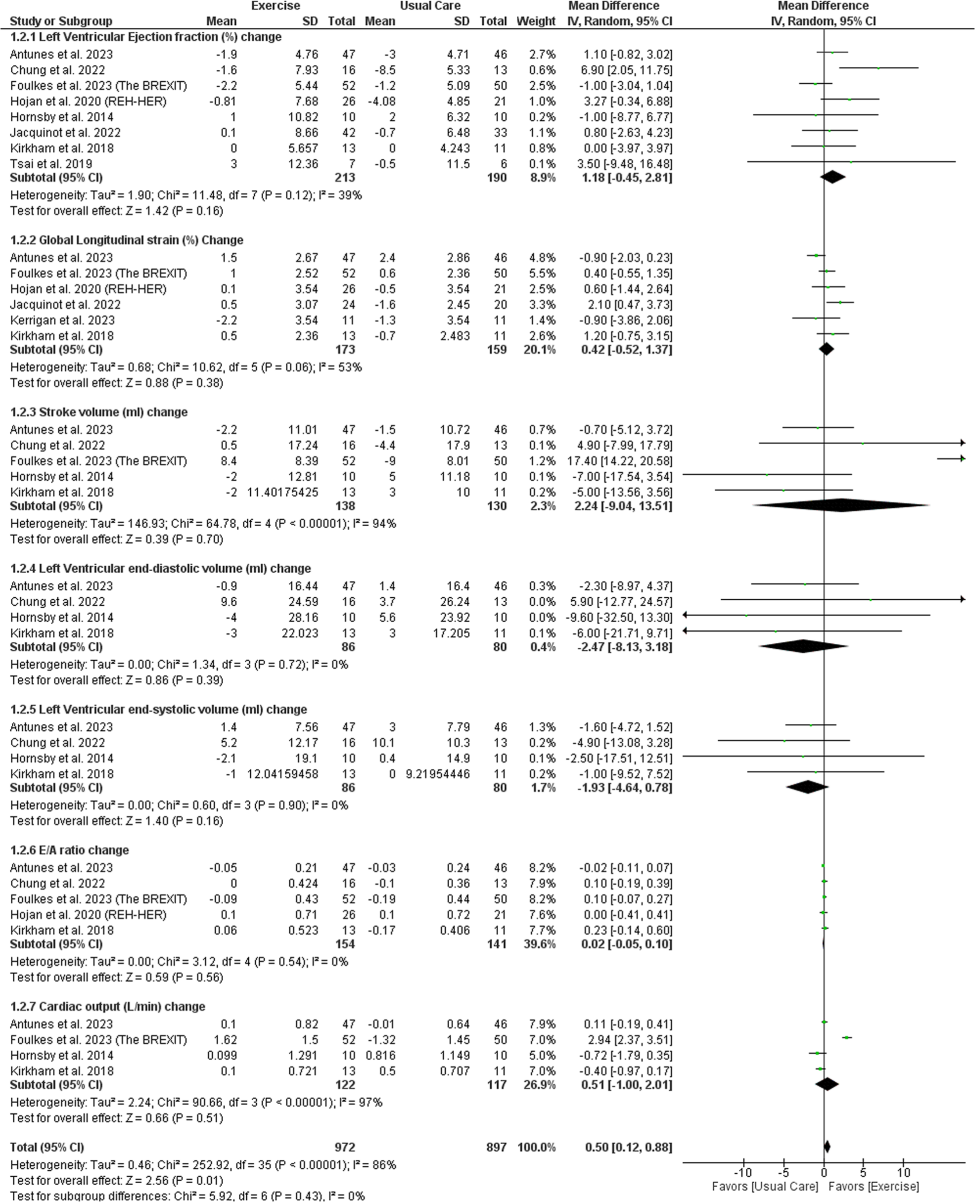
Forest plots of the secondary efficacy outcomes, (1: Left ventricular ejection fraction (LVEF) change, 2: Global longitudinal strain (GLS) change, 3: Stroke volume (SV) change, 4: Left ventricular end-diastolic volume (LVEDV) change, 5: Left ventricular end-systolic volume (LVESV) change, 6: E/A ratio change, and 7: Cardiac output (CO) change), MD: mean difference, CI: confidence interval

There was no significant difference between exercise and usual care regarding LVEF change (MD: 1.18 with 95% CI [-0.45, 2.81], *P* = 0.16), GLS change (MD: 0.42 with 95% CI [-0.52, 1.37], *P* = 0.38), CO change (MD: 0.51 with 95% CI [-1.00, 2.01], *P* = 0.51), SV change (MD: 2.24 with 95% CI [-9.04, 13.51], *P* = 0.70), LVEDV change (MD: -2.47 with 95% CI [-8.13, 3.18], *P* = 0.39), LVESV change (MD: -1.93 with 95% CI [-4.64, 0.78], *P* = 0.16), E/A ratio change (MD: 0.02 with 95% CI [-0.05, 0.10], *P* = 0.56) (Fig. 4).

Moreover, there was no significant difference between exercise and usual care regarding RER change (MD: 0.02 with 95% CI [-0.02, 0.05], *P* = 0.31) (Figure S3), RHR change (MD: -1.63 with 95% CI [-4.64, 1.39], *P* = 0.29) (Figure S4), PHR change (MD: 3.45 with 95% CI [-0.35, 7.25], *P* = 0.08) (Figure S5), RSBP change (MD: -3.32 with 95% CI [-8.79, 2.15], *P* = 0.23) (Figure S6), RDBP change (MD: -2.47 with 95% CI [-6.39, 1.44], *P* = 0.22) (Figure S7).

The pooled studies were homogenous in LVEF change (I^2^ = 39%, *P* = 0.12), LVEDV change (I^2^ = 0%, *P* = 0.72), LVESV change (I^2^ = 0%, *P* = 0.90), E/a ratio change (I^2^ = 0%, *P* = 0.54), RER change (I^2^ = 0%, *P* = 0.75), RHR change (I^2^ = 0%, *P* = 0.78), PHR change (I^2^ = 0%, *P* = 0.97), RSBP change (I^2^ = 0%, *P* = 0.64), and RDBP change (I^2^ = 0%, *P* = 0.66). However, pooled studies were heterogeneous in GLS change (I^2^ = 53%, *P* = 0.06), CO change (I^2^ = 97%, *P* < 0.00001), and SV change (I^2^ = 94%, *P* < 0.00001). Regarding GLS change, heterogeneity was best resolved by excluding Antunes et al. 2023 and Jacquinot et al. 2022 (I^2^ = 19%, *P* = 0.29), (I^2^ = 0%, *P* = 0.44) respectively. Regarding SV change, heterogeneity was best resolved by excluding Foulkes et al. 2023 (The BREXIT) (I^2^ = 0%, *P* = 0.43). Regarding CO change, heterogeneity was best resolved by excluding Foulkes et al. 2023 (The BREXIT) (I^2^ = 45%, *P* = 0.18) (Table S3). The test of subgroup analysis regarding exercise type was insignificant in all the outcomes. The subgroup analysis can be found in (Figures S8-19). Moreover, test for subgroup analysis was not significant regarding whether the patients had breast cancer only or breast cancer plus other cancers (Figure S20-S23).

#### Safety outcomes

**Fig. 5:**
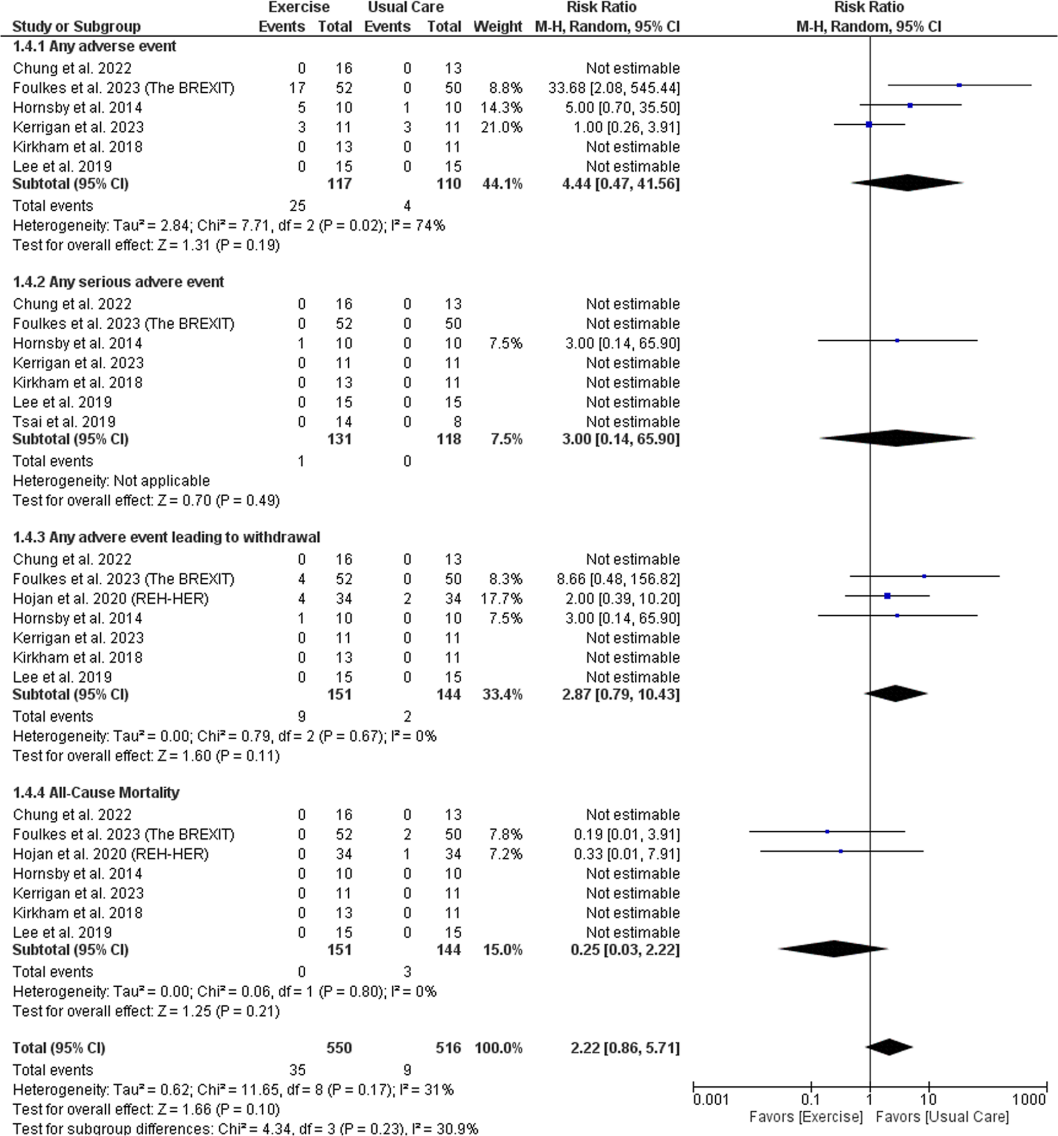
Forest plot of the adverse events, RR: risk ratio, CI: confidence interval

There was no significant difference between exercise and usual care regarding the incidence of any adverse event (RR: 4.44 with 95% CI [0.47, 41.56], *P* = 0.19), any serious adverse event (RR: 3.00 with 95% CI [0.14, 65.90], *P* = 0.49), any adverse event leading to withdrawal (RR: 2.87 with 95% CI [0.79, 10.43], *P* = 0.11), and all-cause mortality (RR: 0.25 with 95% CI [0.03, 2.22], *P* = 0.21) (Fig. 5). Pooled studies were heterogenous in any adverse event (I^2^ = 74%, *P* = 0.02). However, the pooled studies were homogenous in any adverse event leading to withdrawal (I^2^ = 0%, *P* = 0.67) and All-cause mortality (I^2^ = 0%, *P* = 0.80). Regarding any adverse event, heterogeneity was best resolved by excluding Foulkes et al. 2023 (The BREXIT) and Kerrigan et al. 2023 (I^2^ = 45%, *P* = 0.18), (I^2^ = 33%, *P* = 0.22) respectively (Table S3).

## Discussion

This meta-analysis showed that exercise is an effective enhancer of VO_2_ peak in chemotherapy patients. Furthermore, compared to usual care, exercise does not elicit any significant improvement in heart function-related parameters, including LVEF, GLS, CO, SV, LVEDV, LVESV, E/A ratio, RER, RHR, PHR, RSBP, and RDBP. Also, exercise-based care was a tolerable approach during chemotherapy that does not expose any additional risks for adverse events, confirming previous results from the oncology population [33–35].

VO_2_ peak refers to the limited value of oxygen uptake/consumption actually achieved during an exercise test (e.g., running on a treadmill). In other words, VO_2_ peak is the greatest value of the consumed oxygen by an exercising subject independently to his work rate level [36]. Notably, VO_2_ peak is 30% lower in cancer patients compared to age- and sex-matched healthy individuals who do not practice exercise [37]. Thus, it was shown by Jones et al. to be a strong independent predictor of survival among patients with non-small cell lung cancer. Thus, in these patients, the adjusted hazard ratio of all-cause mortality was 0.64 for a VO_2_ peak of 0.96–1.29 L.min − 1 and even lower, reaching 0.56 for a VO_2_ peak of > 1.29 L.min − 1 compared to VO_2_ peak < 0.96 L.min − 1 [38]. This suggests that a moderate increase in VO_2_ peak is beneficial to improve prognosis in the oncology population.

Our findings indicate that exercise can protect against chemotherapy-induced drop in VO_2_ peak, especially since cancer survivors who received neoadjuvant chemotherapy, compared to those who did not receive it, were reported to display a decreased peak VO_2_ per kg by 23% [39]. It is unclear how exercise would induce this effect; however, several mechanisms seem to be involved. The ability of exercise to reduce body mass index (BMI) during chemotherapy was confirmed by a recent systematic review [40]. Therefore, exercise may improve VO_2_ peak among chemotherapy patients by decreasing their BMI, as the latter is negatively associated with VO_2_ peak [41]. Exercise was also found to increase lean mass among cancer survivors, while the absence of exercise favors skeletal muscle loss within the same category [42, 43]. This can contribute to the exercise-induced improvement in cancer-related fatigue in oncology patients as lean mass increase is likely to be accompanied by a VO_2_ peak increase [44].In line with this, results from animal experiment have demonstrated that in rats receiving doxorubicin (a chemotherapy drug known by its toxic effects on skeletal muscle), preconditioning with exercise had enabled the prevention/minimization of skeletal muscle atrophy, contractile dysfunction, and muscular fatigue [45, 46]. Not just that but endurance exercise was shown to reverse doxorubicin-induced myotoxicity in rats [47]. All this may suggest that VO_2_ peak can be boosted in exercising oncology patients by a peripheral mechanism through positive effects on muscular growth, strength, metabolic function and recovery which would ultimately ameliorate oxygen uptake at the local level (muscle VO_2_). Especially that we found no significant benefit of exercise on central (i.e., cardiac) hemodynamics, which makes the peripheral action on skeletal muscle the more likely way to boost VO_2_ peak after chemotherapy. Moreover, higher systemic inflammation is correlated with lower VO_2_ peaks among cancer patients [48], and it is well-established that chemotherapy has pro-inflammatory effects. Therefore, exercise may also elevate VO_2_ peak via its potential to protect cancer survivors from systemic inflammation, particularly chemotherapy [49, 50].

Exercise failed to ameliorate the cardiovascular function of chemotherapy patients, which signifies that training therapy is potentially devoid of substantial protective effects against CIC. The absence of improvement in CO, LVEF, SV, LVEDV, LVESV, GLS, and E/A ratio indicates the inefficacy of exercise in reducing chemotherapy-induced left ventricular dysfunction and heart failure. Moreover, the fact that exercise did not show beneficial chronotropic effects (no changes in RHR and PHR) does not support the protective value of training programs against tachyarrhythmias associated with chemotherapeutic agents. Furthermore, a number of cytotoxic drugs, such as platinum components and alkylating agents, can induce secondary hypertension [51]. The insensibility of RSBP and RDBP to exercise-based therapy shows that the latter may have no notable effects on reducing the susceptibility to chemotherapy-induced hypertension.

It is necessary to determine the safety profile of any intervention among chemotherapy patients due to their vulnerability and frequent comorbidity. Notably, we confirmed in this study that exercise is a tolerable non-pharmacological option during chemotherapy treatment. This is consistent with the findings of a recent meta-analysis, which reported the absence of any harmful effects of exercise on cancer patients undergoing systemic treatment [33]. Another meta-analysis concluded exercise safety and feasibility among colorectal cancer patients [35]. This indicates that chemotherapy survivors may receive exercise-based care without any concerns of harm to reduce the impact of cancer on quality of life (tertiary prevention) and, at the same time, decrease the cardiovascular and metabolic risk in this vulnerable population.

## Strengths and limitations

Few previous meta-analyses have addressed exercise's efficacy and safety profile in preventing CIC [52–54]. However, they either focused on one specific oncology population (i.e., breast cancer patients), one particular chemotherapy agent, or on safety outcomes only. Whereas our study provided a more robust examination of both possible cardiac benefits and harms of training among all oncology chemotherapy survivors. We thoroughly analyzed the available evidence using data from 952 participants and generated important findings about the benefit of exercise on cardiac function and aerobic fitness among cancer survivors managed with chemotherapy.

Nevertheless, our study was prone to considerable limitations as the available data from RCT was incomplete, and the involved studies presented significant heterogeneities and risk of bias concerns that could distort the final interpretations. Additionally, we did not provide a subgroup analysis of different chemotherapeutic agents. Finally, we did not assess the contribution of exercise in altering the susceptibility to develop or exacerbate myocardial ischemia, peripheral artery disease, thromboembolic disease, and myocarditis/pericarditis among chemotherapy patients as the evaluation of these outcomes would require other biomarkers (troponin elevation, ECG changes, INR drop for patients taking anticoagulants, vascular imaging, etc.), which are not included in our study.

## Implications and future perspectives

The cardiovascular complications of cytotoxic molecules regroup a large spectrum of diseases [2]. Our results demonstrated a very modest benefit of exercise on the cardiac function of patients receiving chemotherapeutic agents, thereby, its low suitability to counteract chemotherapy-induced heart dysfunction. However, there is a potential for other cardioprotective effects not evaluated in our study, such as anti-ischemic, anti-thrombotic, and anti-inflammatory effects on chemotherapy-exposed cardiovascular tissue. Hence, future research should analyze the preventive abilities of physical activity against CIC events that may not necessarily lead to altered cardiac function, such as ischemic heart disease, peripheral artery disease, venous thromboembolism, and inflammatory reactions of the heart layers (myocarditis, pericarditis). On the other hand, the findings of our study suggest that there is a need for effective pharmacological and non-pharmacological strategies to prevent the decline in cardiac function secondary to chemotherapy. The only medication approved by the United States Food and Drug Administration (FDA) and European Medicines Agency (EMA) to prevent anthracycline-related cardiomyopathy is dexrazoxane [55]. However, other treatments were also found to be effective in preventing CIC, such as statins, beta-blockers, angiotensin-converting enzyme inhibitors, and aldosterone receptor antagonists, particularly spironolactone [56]. Therefore, the effectiveness of such therapies should be further investigated, and once confirmed, they may be approved for clinical use. The good tolerability of physical training programs by chemotherapy patients should motivate more investigation about the other possible benefits of this type of care apart from enhancing cardiovascular function and preventing CIC.

## Conclusion

Exercise has limited beneficial effects on cardiac function among chemotherapy patients, manifesting mainly as a relative boosting of aerobic fitness. Nevertheless, it is a safe and tolerable strategy that may hold other interesting advantages to cancer survivors worthy of investigation. Moreover, the fact that exercise did not show beneficial chronotropic effects (no changes in RHR and PHR) does not support the protective value of training programs against tachyarrhythmias associated with chemotherapeutic agents. The absence of improvement in CO, LVEF, SV, LVEDV, LVESV, GLS, and E/A ratio indicates the inefficacy of exercise in reducing chemotherapy-induced left ventricular dysfunction and heart failure. Despite the shown lack of proof of effectiveness, future studies should still search for any possible cardioprotective potentials of physical training during chemotherapy. Parallel to this, it is also necessary to identify pharmacological or non-pharmacological strategies other than exercise to antagonize the cardiovascular harms of different chemotherapeutic drugs effectively.

### Supplementary Information

**Additional file 1: Table S1.** Search strategy. **Table S2.** Authors' description of risk of bias assessment. **Table S3.** Sensitivity analysis. **Figure S1.** VO_2_ peak subgroup analysis based on exercise type. **Figure S2.** VO_2_ peak subgroup analysis based on whether the patients had breast cancer only or breast cancer plus other cancers. **Figure S3.** Forest plot of respiratory exchange ratio (RER) change. **Figure S4.** Forest plot of resting heart rate (RHR) change. **Figure S5.** Forest plot of peak heart rate (PHR) change. **Figure S6.** Forest plot of resting systolic blood pressure (RSBP) change. **Figure S7.** Forest plot of resting diastolic blood pressure (RDBP) change. **Figure S8.** Left ventricular ejection fraction (LVEF) subgroup analysis based on exercise type. **Figure S9.** Cardiac output (CO) subgroup analysis based on exercise type. **Figure S10.** E/a ratio subgroup analysis based on exercise type. **Figure S11.** Global longitudinal strain (GLS) subgroup analysis based on exercise type. **Figure S12.** Left ventricular end-systolic volume (LVESV) subgroup analysis based on exercise type. **Figure S13.** Left ventricular end-diastolic volume (LVEDV) subgroup analysis based on exercise type. **Figure S14.** Resting heart rate (RHR) subgroup analysis based on exercise type. **Figure S15.** Peak heart rate (PHR) subgroup analysis based on exercise type. **Figure S16.** Respiratory exchange ratio (RER) subgroup analysis based on exercise type. **Figure S17.** Resting systolic blood pressure (RSBP) subgroup analysis based on exercise type. **Figure S18.** Resting diastolic blood pressure (RDBP) subgroup analysis based on exercise type. **Figure S19.** Stroke volume (SV) subgroup analysis based on exercise type. **Figure S20.** Left ventricular ejection fraction (LVEF) subgroup analysis based on whether the patients had breast cancer only or breast cancer plus other cancers. **Figure S21.** Global longitudinal strain (GLS) subgroup analysis based on whether the patients had breast cancer only or breast cancer plus other cancers. **Figure S22.** Respiratory exchange ratio (RER) subgroup analysis based on whether the patients had breast cancer only or breast cancer plus other cancers. **Figure S23.** Peak heart rate (PHR) subgroup analysis based on whether the patients had breast cancer only or breast cancer plus other cancers.

## Data Availability

Not applicable.

## Abbreviations

CIC: Chemotherapy-induced cardiotoxicity
CAD: Coronary artery disease
ROS: Reactive oxygen species
TSMB: Trial sequential monitoring boundary
TSA: Trial Sequential Analysis
GRADE: Grading of Recommendations Assessment, Development, and Evaluation criteria
LVEF: Left ventricular ejection fraction
GLS: Global longitudinal strain
LVEDV: Left ventricular end-diastolic volume
LVESV: Left ventricular end-systolic volume
5-FU: 5-Fluorouracil
CO: Cardiac output
SV: Stroke volume
RER: Respiratory exchange ratio
RHR: Resting heart rate
PHR: Peak heart rate
RDBP: Resting diastolic blood pressure
RSBP: Resting systolic blood pressure
