# Supplementary material for: The efficacy and safety of exercise regimens to mitigate chemotherapy cardiotoxicity: a systematic review and meta-analysis of randomized controlled trials
Source: Cardiooncology. 2024 Feb 23;10:10. doi: 10.1186/s40959-024-00208-2 (PMC10885653; doi:10.1186/s40959-024-00208-2)
Supplement: Supplementary file 1 — Additional file 1: Table S1. Search strategy. Table S2. Authors' description of risk of bias assessment. Table S3. Sensitivity analysis. Figure S1. VO2 peak subgroup analysis based on exercise type. Figure S2. VO2 peak subgroup analysis based on whether the patients had breast cancer only or breast cancer plus other cancers. Figure S3. Forest plot of respiratory exchange ratio (RER) change. Figure S4. Forest plot of resting heart rate (RHR) change. Figure S5. Forest plot of peak heart rate (PHR) change. Figure S6. Forest plot of resting systolic blood pressure (RSBP) change. Figure S7. Forest plot of resting diastolic blood pressure (RDBP) change. Figure S8. Left ventricular ejection fraction (LVEF) subgroup analysis based on exercise type. Figure S9. Cardiac output (CO) subgroup analysis based on exercise type. Figure S10. E/a ratio subgroup analysis based on exercise type. Figure S11. Global longitudinal strain (GLS) subgroup analysis based on exercise type. Figure S12. Left ventricular end-systolic volume (LVESV) subgroup analysis based on exercise type. Figure S13. Left ventricular end-diastolic volume (LVEDV) subgroup analysis based on exercise type. Figure S14. Resting heart rate (RHR) subgroup analysis based on exercise type. Figure S15. Peak heart rate (PHR) subgroup analysis based on exercise type. Figure S16. Respiratory exchange ratio (RER) subgroup analysis based on exercise type. Figure S17. Resting systolic blood pressure (RSBP) subgroup analysis based on exercise type. Figure S18. Resting diastolic blood pressure (RDBP) subgroup analysis based on exercise type. Figure S19. Stroke volume (SV) subgroup analysis based on exercise type. Figure S20. Left ventricular ejection fraction (LVEF) subgroup analysis based on whether the patients had breast cancer only or breast cancer plus other cancers. Figure S21. Global longitudinal strain (GLS) subgroup analysis based on whether the patients had breast cancer only or breast cancer plus other cancers. Figure S2 [file 40959_2024_208_MOESM1_ESM.docx]

**Supplementary material:**

**Title.**

**Efficacy and safety of exercise on cancer patients receiving cardiotoxic chemotherapeutic drugs: A Systematic Review and Meta-analysis of Randomized Controlled Trials.**

**Running Title.**

Exercise for cancer patients receiving cardiotoxic drugs.

**Authors.**

Ahmed Mazen Amin^1^, Yehya Khlidj^2^, Mohamed Abuelazm^3^, Ahmed A. Ibrahim^4^, Mohammad Tanashat^5^, Muhammad Imran^6^, Abubakar Nazir^7^, Hosam Shaikhkhalil^8^, Basel Abdelazeem^9^.

**Affiliations.**

1. Faculty of Medicine, Mansoura University, Mansoura, Egypt.
2. Faculty of Medicine, Algiers University, Algiers, Algeria.
3. Faculty of Medicine, Tanta University, Tanta, Egypt.
4. Faculty of Medicine, Menoufia University, Menoufia, Egypt.
5. Faculty of Medicine, Yarmouk University, Irbid, Jordan.
6. University College of Medicine and Dentistry, The University of Lahore, Lahore, Pakistan.
7. Faculty of Medicine, King Edward Medical University, Lahore, Pakistan.
8. Faculty of Medicine, Islamic University of Gaza, Gaza, Palestine.
9. Department of Cardiology, West Virginia University, West Virginia, USA.

**Keywords.**

Exercise; Cancer; Chemotherapy; Cardiotoxic; Review; Meta-analysis

**Contents:**

**Tables.**Table S1: Search strategy.

Table S2: Authors' description of risk of bias assessment.

Table S3: Sensitivity analysis.

**Figures.**

Figure S1: VO_2_ peak subgroup analysis based on exercise type.

Figure S2: VO_2_ peak subgroup analysis based on whether the patients had breast cancer only or breast cancer plus other cancers.

Figure S3: Forest plot of respiratory exchange ratio (RER) change.

Figure S4: Forest plot of resting heart rate (RHR) change.

Figure S5: Forest plot of peak heart rate (PHR) change.

Figure S6: Forest plot of resting systolic blood pressure (RSBP) change.

Figure S7: Forest plot of resting diastolic blood pressure (RDBP) change.

Figure S8: Left ventricular ejection fraction (LVEF) subgroup analysis based on exercise type.

Figure S9: Cardiac output (CO) subgroup analysis based on exercise type.

Figure S10: E/a ratio subgroup analysis based on exercise type.

Figure S11: Global longitudinal strain (GLS) subgroup analysis based on exercise type.

Figure S12: Left ventricular end-systolic volume (LVESV) subgroup analysis based on exercise type.

Figure S13: Left ventricular end-diastolic volume (LVEDV) subgroup analysis based on exercise type.

Figure S14: Resting heart rate (RHR) subgroup analysis based on exercise type.

Figure S15: Peak heart rate (PHR) subgroup analysis based on exercise type.

Figure S16: Respiratory exchange ratio (RER) subgroup analysis based on exercise type.

Figure S17: Resting systolic blood pressure (RSBP) subgroup analysis based on exercise type.

Figure S18: Resting diastolic blood pressure (RDBP) subgroup analysis based on exercise type.

Figure S19: Stroke volume (SV) subgroup analysis based on exercise type.

Figure S20: Left ventricular ejection fraction (LVEF) subgroup analysis based on whether the patients had breast cancer only or breast cancer plus other cancers.

Figure S21: Global longitudinal strain (GLS) subgroup analysis based on whether the patients had breast cancer only or breast cancer plus other cancers.

Figure S22: Respiratory exchange ratio (RER) subgroup analysis based on whether the patients had breast cancer only or breast cancer plus other cancers.

Figure S23: Peak heart rate (PHR) subgroup analysis based on whether the patients had breast cancer only or breast cancer plus other cancers.

**Concept1:**

(Exercise* OR "Physical Activit*" OR walking OR sport* OR training OR Kinesiotherapy OR kinesiatrics OR "Cardiac Rehabilitation")

**Concept2:**

(Anthracycline* OR Mitoxantrone OR Chemotherap* OR Doxorubicin OR Aclarubicin OR Adriamycin OR Carubicin OR DOX OR Daunorubicin OR Cerubidine OR Idarubicin OR Idamycin OR Epirubicin OR Ellence OR Novantrone OR Nogalamycin OR Plicamycin OR Fluorouracil OR Capecitabine OR "5-FU" OR "5-fluorouracil" OR Trastuzumab OR pertuzumab OR Lapatinib OR Sunitinib OR Sorafenib OR Imatinib OR dasatinib OR Nilotinib OR Ibrutinib OR nivolumab OR Pembrolizumab OR atezolizumab OR Ipilimumab)

**Concept 3:**

(cardiotoxicit* OR cardiomyopath* OR cardiotoxic OR CTRCD OR "Cardiac Failure" OR "Cardiac dysfunction" OR "Cardiac damage" OR "Cardiac myopathy" OR "cardiac apoptosis" OR "heart failure" OR "heart toxicity" OR "heart damage" OR "heart dysfunction" OR "myocardial failure" OR "Myocardial toxicity" OR "myocardial damage" OR "myocardial dysfunction" OR "ventricular failure" OR "ventricular toxicity" OR "ventricular damage" OR "cardiomyocyte* damage" OR "cardiomyocyte* toxicity" OR "cardiomyocyte* dysfunction" OR "cardiomyocyte* apoptosis" OR "cardiac injury")

__________________________________________________________________________________________________________________________

Date: 14-7-2023

| Database | Search Terms | Search Field | Search Results |
| --- | --- | --- | --- |
| Pubmed | ((Exercise* OR "Physical Activit*" OR walking OR sport* OR training OR Kinesiotherapy OR kinesiatrics OR "Cardiac Rehabilitation") AND (Anthracycline* OR Mitoxantrone OR Chemotherap* OR Doxorubicin OR Aclarubicin OR Adriamycin OR Carubicin OR DOX OR Daunorubicin OR Cerubidine OR Idarubicin OR Idamycin OR Epirubicin OR Ellence OR Novantrone OR Nogalamycin OR Plicamycin OR Fluorouracil OR Capecitabine OR "5-FU" OR "5-fluorouracil" OR Trastuzumab OR pertuzumab OR Lapatinib OR Sunitinib OR Sorafenib OR Imatinib OR dasatinib OR Nilotinib OR Ibrutinib OR nivolumab OR Pembrolizumab OR atezolizumab OR Ipilimumab) AND (cardiotoxicit* OR cardiomyopath* OR cardiotoxic OR CTRCD OR "Cardiac Failure" OR "Cardiac dysfunction" OR "Cardiac damage" OR "Cardiac myopathy" OR "cardiac apoptosis" OR "heart failure" OR "heart toxicity" OR "heart damage" OR "heart dysfunction" OR "myocardial failure" OR "Myocardial toxicity" OR "myocardial damage" OR "myocardial dysfunction" OR "ventricular failure" OR "ventricular toxicity" OR "ventricular damage" OR "cardiomyocyte* damage" OR "cardiomyocyte* toxicity" OR "cardiomyocyte* dysfunction" OR "cardiomyocyte* apoptosis" OR "cardiac injury")) | All Field | 999 |
| Cochrane | ((Exercise* OR (Physical NEXT Activit*) OR walking OR sport* OR training OR Kinesiotherapy OR kinesiatrics OR "Cardiac Rehabilitation") AND (Anthracycline* OR Mitoxantrone OR Chemotherap* OR Doxorubicin OR Aclarubicin OR Adriamycin OR Carubicin OR DOX OR Daunorubicin OR Cerubidine OR Idarubicin OR Idamycin OR Epirubicin OR Ellence OR Novantrone OR Nogalamycin OR Plicamycin OR Fluorouracil OR Capecitabine OR "5-FU" OR "5-fluorouracil" OR Trastuzumab OR pertuzumab OR Lapatinib OR Sunitinib OR Sorafenib OR Imatinib OR dasatinib OR Nilotinib OR Ibrutinib OR nivolumab OR Pembrolizumab OR atezolizumab OR Ipilimumab) AND (cardiotoxicit* OR cardiomyopath* OR cardiotoxic OR CTRCD OR "Cardiac Failure" OR "Cardiac dysfunction" OR "Cardiac damage" OR "Cardiac myopathy" OR "cardiac apoptosis" OR "heart failure" OR "heart toxicity" OR "heart damage" OR "heart dysfunction" OR "myocardial failure" OR "Myocardial toxicity" OR "myocardial damage" OR "myocardial dysfunction" OR "ventricular failure" OR "ventricular toxicity" OR "ventricular damage" OR (cardiomyocyte* NEXT damage) OR (cardiomyocyte* NEXT toxicity) OR (cardiomyocyte* NEXT dysfunction) OR (cardiomyocyte* AND apoptosis) OR "cardiac injury")) | All Field | 144 |
| WOS | ((Exercise* OR "Physical Activit*" OR walking OR sport* OR training OR Kinesiotherapy OR kinesiatrics OR "Cardiac Rehabilitation") AND (Anthracycline* OR Mitoxantrone OR Chemotherap* OR Doxorubicin OR Aclarubicin OR Adriamycin OR Carubicin OR DOX OR Daunorubicin OR Cerubidine OR Idarubicin OR Idamycin OR Epirubicin OR Ellence OR Novantrone OR Nogalamycin OR Plicamycin OR Fluorouracil OR Capecitabine OR "5-FU" OR "5-fluorouracil" OR Trastuzumab OR pertuzumab OR Lapatinib OR Sunitinib OR Sorafenib OR Imatinib OR dasatinib OR Nilotinib OR Ibrutinib OR nivolumab OR Pembrolizumab OR atezolizumab OR Ipilimumab) AND (cardiotoxicit* OR cardiomyopath* OR cardiotoxic OR CTRCD OR "Cardiac Failure" OR "Cardiac dysfunction" OR "Cardiac damage" OR "Cardiac myopathy" OR "cardiac apoptosis" OR "heart failure" OR "heart toxicity" OR "heart damage" OR "heart dysfunction" OR "myocardial failure" OR "Myocardial toxicity" OR "myocardial damage" OR "myocardial dysfunction" OR "ventricular failure" OR "ventricular toxicity" OR "ventricular damage" OR "cardiomyocyte* damage" OR "cardiomyocyte* toxicity" OR "cardiomyocyte* dysfunction" OR "cardiomyocyte* apoptosis" OR "cardiac injury")) | All Field | 1274 |
| SCOPUS | TITLE-ABS-KEY ((Exercise* OR "Physical Activit*" OR walking OR sport* OR training OR Kinesiotherapy OR kinesiatrics OR "Cardiac Rehabilitation") AND (Anthracycline* OR Mitoxantrone OR Chemotherap* OR Doxorubicin OR Aclarubicin OR Adriamycin OR Carubicin OR DOX OR Daunorubicin OR Cerubidine OR Idarubicin OR Idamycin OR Epirubicin OR Ellence OR Novantrone OR Nogalamycin OR Plicamycin OR Fluorouracil OR Capecitabine OR "5-FU" OR "5-fluorouracil" OR Trastuzumab OR pertuzumab OR Lapatinib OR Sunitinib OR Sorafenib OR Imatinib OR dasatinib OR Nilotinib OR Ibrutinib OR nivolumab OR Pembrolizumab OR atezolizumab OR Ipilimumab) AND (cardiotoxicit* OR cardiomyopath* OR cardiotoxic OR CTRCD OR "Cardiac Failure" OR "Cardiac dysfunction" OR "Cardiac damage" OR "Cardiac myopathy" OR "cardiac apoptosis" OR "heart failure" OR "heart toxicity" OR "heart damage" OR "heart dysfunction" OR "myocardial failure" OR "Myocardial toxicity" OR "myocardial damage" OR "myocardial dysfunction" OR "ventricular failure" OR "ventricular toxicity" OR "ventricular damage" OR "cardiomyocyte* damage" OR "cardiomyocyte* toxicity" OR "cardiomyocyte* dysfunction" OR "cardiomyocyte* apoptosis" OR "cardiac injury")) | Title, Abstract, Keywords | 1332 |
| EMBASE | #4. #1 AND #2 AND #3 655  #3. cardiotoxicit*:ti,ab,kw OR 553,143  cardiomyopath*:ti,ab,kw OR cardiotoxic:ti,ab,kw  OR ctrcd:ti,ab,kw OR 'cardiac failure':ti,ab,kw  OR 'cardiac dysfunction':ti,ab,kw OR 'cardiac  damage':ti,ab,kw OR 'cardiac myopathy':ti,ab,kw  OR 'cardiac apoptosis':ti,ab,kw OR 'heart  failure':ti,ab,kw OR 'heart toxicity':ti,ab,kw OR  'heart damage':ti,ab,kw OR 'heart  dysfunction':ti,ab,kw OR 'myocardial  failure':ti,ab,kw OR 'myocardial  toxicity':ti,ab,kw OR 'myocardial  damage':ti,ab,kw OR 'myocardial  dysfunction':ti,ab,kw OR 'ventricular  failure':ti,ab,kw OR 'ventricular  toxicity':ti,ab,kw OR 'ventricular  damage':ti,ab,kw OR 'cardiomyocyte*  damage':ti,ab,kw OR 'cardiomyocyte*  toxicity':ti,ab,kw OR 'cardiomyocyte*  dysfunction':ti,ab,kw OR 'cardiomyocyte*  apoptosis':ti,ab,kw OR 'cardiac injury':ti,ab,kw  #2. anthracycline:ti,ab,kw OR mitoxantrone:ti,ab,kw 997,873  OR chemotherap*:ti,ab,kw OR doxorubicin:ti,ab,kw  OR aclarubicin:ti,ab,kw OR adriamycin:ti,ab,kw OR  carubicin:ti,ab,kw OR daunorubicin:ti,ab,kw OR  cerubidine:ti,ab,kw OR idarubicin:ti,ab,kw OR  idamycin:ti,ab,kw OR epirubicin:ti,ab,kw OR  ellence:ti,ab,kw OR novantrone:ti,ab,kw OR  nogalamycin:ti,ab,kw OR plicamycin:ti,ab,kw OR  fluorouracil:ti,ab,kw OR capecitabine:ti,ab,kw OR  '5 fluorouracil':ti,ab,kw OR trastuzumab:ti,ab,kw  OR pertuzumab:ti,ab,kw OR lapatinib:ti,ab,kw OR  sunitinib:ti,ab,kw OR sorafenib:ti,ab,kw OR  imatinib:ti,ab,kw OR dasatinib:ti,ab,kw OR  nilotinib:ti,ab,kw OR ibrutinib:ti,ab,kw OR  nivolumab:ti,ab,kw OR pembrolizumab:ti,ab,kw OR  atezolizumab:ti,ab,kw OR ipilimumab:ti,ab,kw  #1. exercise*:ti,ab,kw OR 'physical 1,475,211  activit*':ti,ab,kw OR walking:ti,ab,kw OR  sport*:ti,ab,kw OR training:ti,ab,kw OR  kinesiotherapy:ti,ab,kw OR kinesiatrics:ti,ab,kw  OR 'heart rehabilitation':ti,ab,kw | All Field | 655 |
| Clinical trials.gov | Exercise AND cardiotoxicity | All Field | 23 |
| MedRxiv | Exercise AND cardiotoxicity | All Field | 17 |

Table S1: Search Strategy.

| Study | Decision | description |
| --- | --- | --- |
| Lee et al 2019 | Some concerns | There is no information if the allocation sequence was concealed until participants were enrolled and assigned to the intervention and the assessment of the outcome has been influenced by knowledge of the intervention received |
| Sturgeon et al 2022 | Some concerns | There is no information if the allocation sequence was concealed until participants were enrolled and assigned to the intervention and the assessment of the outcome has been influenced by knowledge of the intervention received |
| Kirkham et al 2018 | Some concerns | The assessment of the outcome has been influenced by knowledge of the intervention received |
| Tsai et al 2019 | High risk | The allocation sequence was not randomized in all patients, and there is no information if an appropriate analysis was used to estimate the effect of assignment to intervention. |
| Hojan et al 2020 | Some concerns | There is no information if their non-adherence to the assigned intervention regimen could have affected participants' outcomes. |

*Table S2: Authors' description of risk of bias assessment.*

| Outcome | No. of  Participants (/) | No. of  trials | Quantitative data synthesis | | | | Heterogeneity analysis | | |
| --- | --- | --- | --- | --- | --- | --- | --- | --- | --- |
|  |  |  | MD | 95% CI | Z value | p-value | df | p-value | I2 (%) |
| **VO2peak, ml/kg/min Change.** | | | | | | | | | |
| Omitting Antunes et al. 2023 | 430/254 | 7 | 1.77 | [0.33, 3.20] | 2.42 | 0.02 | 6 | <0.001 | 89% |
| Omitting Bolam et al.2019 (OptiTrain) | 331/240 | 7 | 2.37 | [1.34, 3.40] | 4.52 | <0.001 | 6 | 0.02 | 60% |
| Omitting Chung et al. 2022 | 461/287 | 7 | 2.22 | [0.77, 3.68] | 2.99 | 0.003 | 6 | <0.001 | 91% |
| Omitting Courneya et al. 2007 | 317/218 | 7 | 2.13 | [0.49, 3.78] | 2.54 | 0.01 | 6 | <0.001 | 90% |
| Omitting Foulkes et al. 2023 (The BREXIT) | 425/250 | 7 | 1.80 | [0.49, 3.11] | 2.69 | 0.007 | 6 | <0.001 | 85% |
| Omitting Hornsby et al. 2014 | 467/290 | 7 | 1.70 | [0.30, 3.10] | 2.39 | 0.02 | 6 | <0.001 | 89% |
| Omitting Jacquinot et al. 2022 | 442/272 | 7 | 1.88 | [0.35, 3.41] | 2.41 | 0.02 | 6 | <0.001 | 88% |
| Omitting Kerrigan et al. 2023 | 466/289 | 7 | 1.93 | [0.52, 3.33] | 2.92 | 0.007 | 6 | <0.001 | 91% |
|  |  |  |  |  |  |  |  |  |  |
| **Global Longitudinal Strain (%) Change.** | | | | | | | | | |
| **Omitting Antunes et al. 2023** | 126/113 | 5 | 0.59 | [-0.21, 1.38] | 145 | 0.15 | 4 | 0.29 | 19% |
| Omitting Foulkes et al. 2023 (The BREXIT) | 121/109 | 5 | 0.21 | [-0.98, 1.41] | 0.35 | 0.73 | 4 | 0.05 | 53% |
| Omitting Hojan et al. 2020 (REH-HER) | 147/138 | 5 | 0.20 | [-0.82, 1.21] | 0.38 | 0.70 | 4 | 0.05 | 58% |
| **Omitting Jacquinot et al. 2022** | 149/139 | 5 | -0.08 | [-0.70, 0.53] | 0.27 | 0.79 | 4 | 0.44 | 0% |
| Omitting Kerrigan et al. 2023 | 162/142 | 5 | 0.34 | [-0.61, 1.29] | 0.70 | 0.48 | 4 | 0.06 | 58% |
| Omitting Kirkham et al. 2018 | 160/148 | 5 | 0.30 | [-0.77, 1.37] | 0.56 | 0.58 | 4 | 0.05 | 59% |
| **Stroke volume (ml) change.** | | | | | | | | | |
| Omitting Antunes et al. 2023 | 91/84 | 4 | 2.93 | [-11.57,17.44] | 0.54 | 0.59 | 3 | <0.001 | 92% |
| Omitting Chung et al. 2022 | 122/117 | 4 | 1.63 | [-11.30,14.57] | 0.39 | 0.80 | 3 | <0.001 | 95% |
| **Omitting Foulkes et al. 2023 (The BREXIT)** | 86/80 | 4 | -1.72 | [-5.26, 1.82] | 0.95 | 0.34 | 3 | 0.43 | 0% |
| Omitting Hornsby et al. 2014 | 128/120 | 4 | 4.38 | [-8.00, 16.76] | 0.69 | 0.49 | 3 | <0.001 | 95% |
| Omitting Kirkham et al. 2018 | 125/119 | 4 | 4.03 | [-8.62, 16.68] | 0.62 | 0.53 | 3 | <0.001 | 94% |
|  |  |  |  |  |  |  |  |  |  |
| **Cardiac output (L/min) change.** | | | | | | | | | |
| Omitting Antunes et al. 2023 | 75/71 | 4 | 0.63 | [-1.85, 3.11] | 0.52 | 0.62 | 2 | <0.001 | 97% |
| **Omitting Foulkes et al. 2023 (The BREXIT)** | 70/67 | 4 | -0.18 | [-0.65, 0.29] | 0.76 | 0.45 | 2 | 0.13 | 50% |
| Omitting Hornsby et al. 2014 | 112/107 | 4 | 0.88 | [-0.91, 2.66] | 0.96 | 0.34 | 2 | <0.001 | 98% |
| Omitting Kirkham et al. 2018 | 109/106 | 4 | 0.80 | [-1.29, 2.90] | 0.75 | 0.45 | 2 | <0.001 | 97% |
|  |  |  |  |  |  |  |  |  |  |
| **Any adverse event.** RR | | | | | | | | | |
| Omitting Chung et al. 2022 | 101/97 | 5 | 4.44 | [0.47, 41.56] | 1.31 | 0.19 | 2 | 0.02 | 74% |
| **Omitting Foulkes et al. 2023 (The BREXIT)** | 65/60 | 5 | 1.92 | [0.39, 9.38] | 0.81 | 0.42 | 2 | 0.18 | 45% |
| Omitting Hornsby et al. 2014 | 107/100 | 5 | 5.06 | [0.07, 385.10] | 0.73 | 0.46 | 2 | 0.005 | 87% |
| **Omitting Kerrigan et al. 2023** | 106/99 | 5 | 10.47 | [1.37, 80.00] | 2.26 | 0.02 | 2 | 0.22 | 33% |
| Omitting Kirkham et al. 2018 | 104/99 | 5 | 4.44 | [0.47, 41.56] | 1.31 | 0.19 | 2 | 0.02 | 74% |
| Lee et al. 2019 | 102/95 | 5 | 4.44 | [0.47, 41.56] | 1.31 | 0.19 | 2 | 0.02 | 74% |

Table S3: Sensitivity analysis.

MD: mean difference; CI: confidence interval; df: degrees of freedom; RR: risk ratio


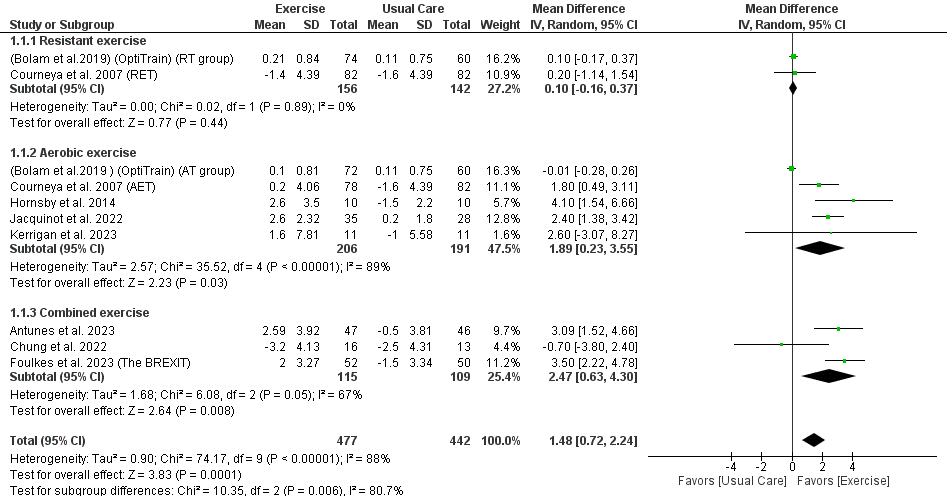


Figure S1: VO_2_ peak subgroup analysis based on exercise type.


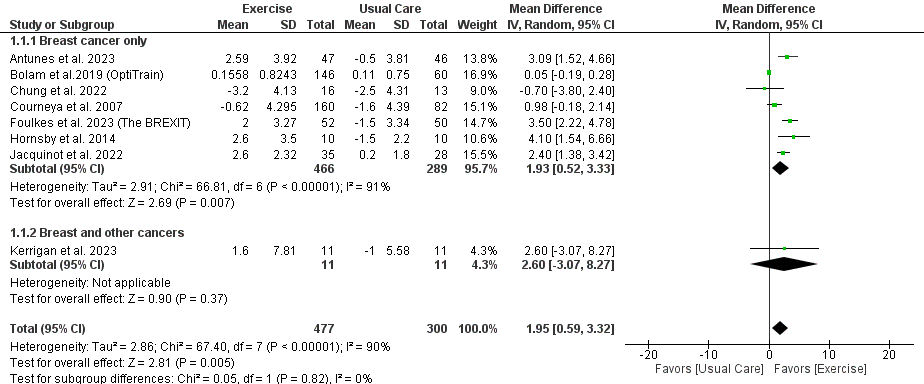


Figure S2: VO_2_ peak subgroup analysis based on whether the patients had breast cancer only or breast cancer plus other cancers.


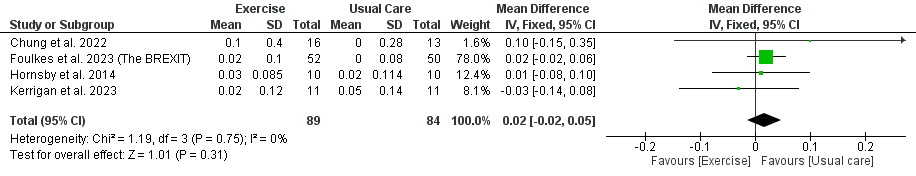


Figure S3: Forest plot of respiratory exchange ratio (RER) change.


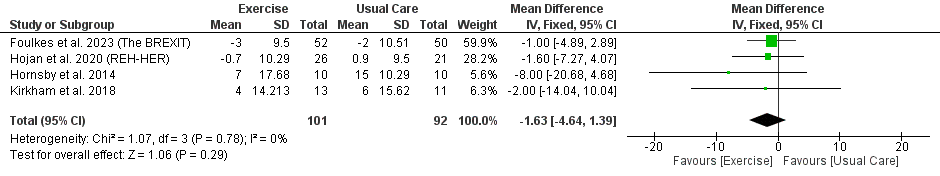


Figure S4: Forest plot of resting heart rate (RHR) change.


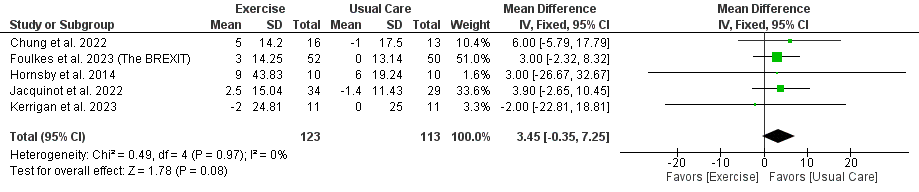


Figure S5: Forest plot of peak heart rate (PHR) change.


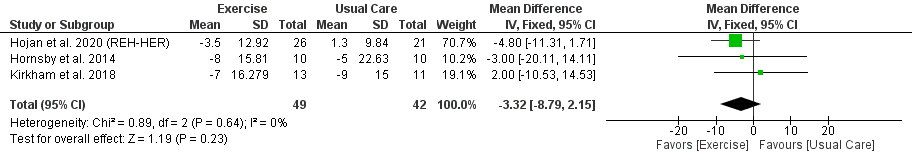


Figure S6: Forest plot of resting systolic blood pressure (RSBP) change.


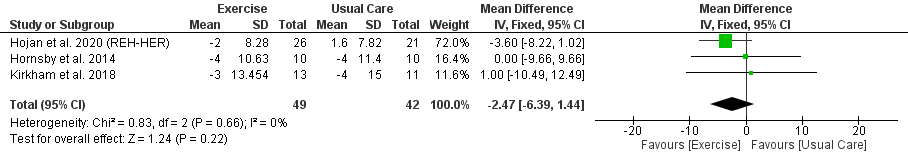


Figure S7: Forest plot of resting diastolic blood pressure (RDBP) change.


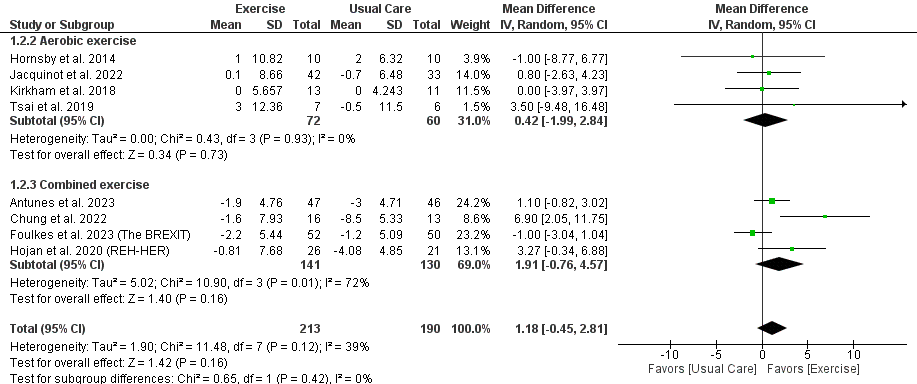


Figure S8: Left ventricular ejection fraction (LVEF) subgroup analysis based on exercise type.


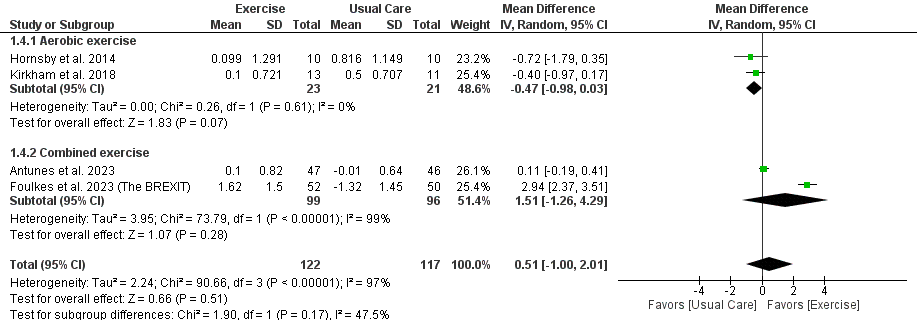


Figure S9: Cardiac output (CO) subgroup analysis based on exercise type.


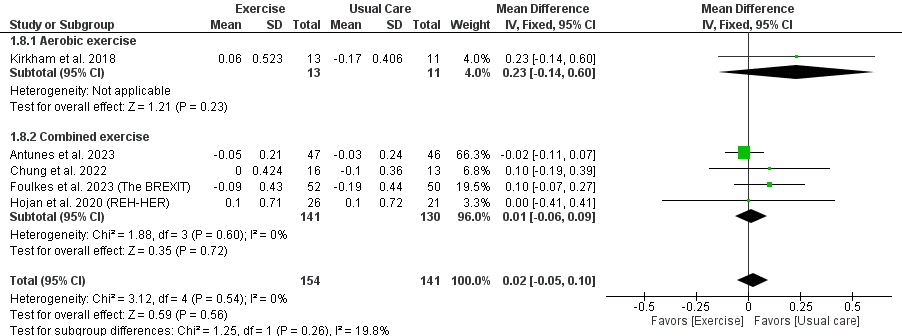


Figure S10: E/a ratio subgroup analysis based on exercise type.


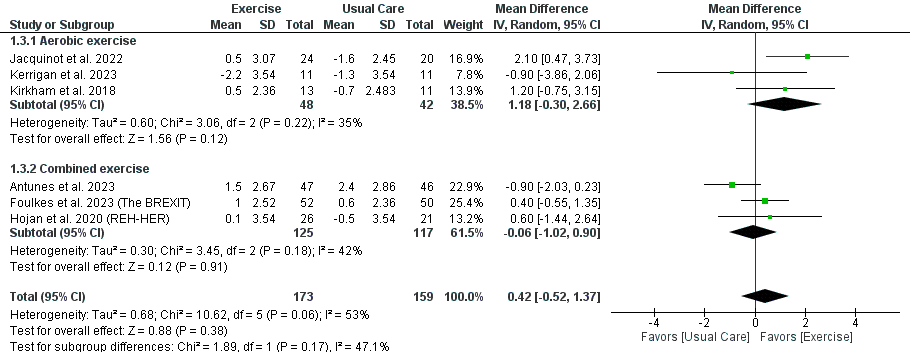


Figure S11: Global longitudinal strain (GLS) subgroup analysis based on exercise type.


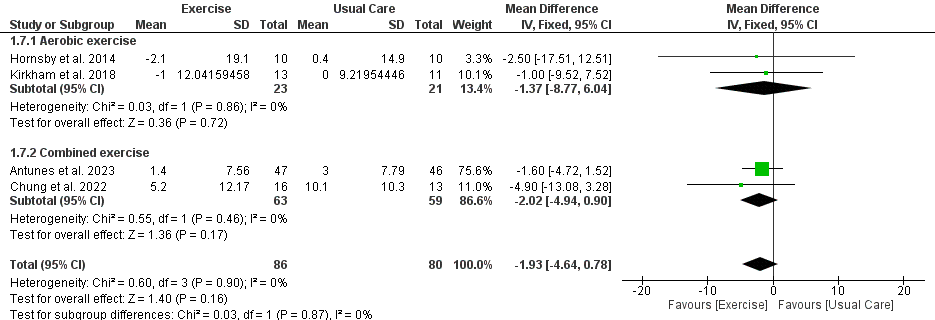


Figure S12: Left ventricular end-systolic volume (LVESV) subgroup analysis based on exercise type.


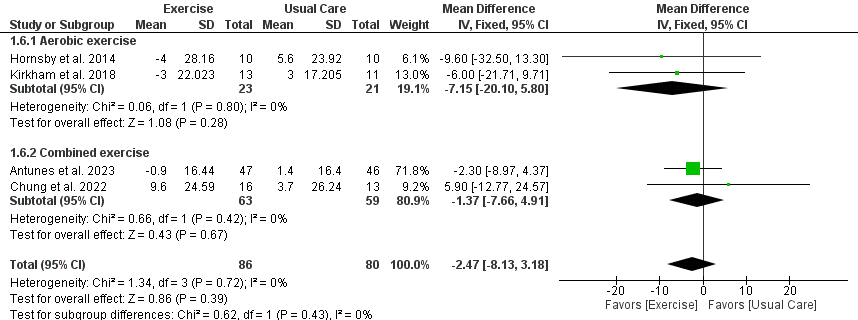


Figure S13: Left ventrciluar end-diastolic volume (LVEDV) subgroup analysis based on exercise type.


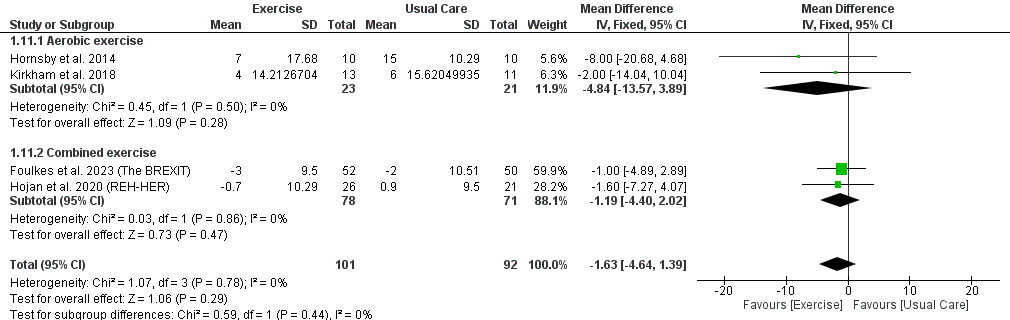


Figure S14: Resting heart rate (RHR) subgroup analysis based on exercise type.


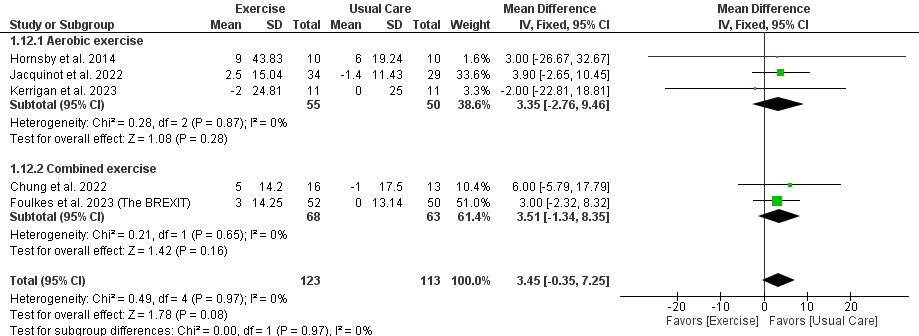


Figure S15: Peak heart rate (PHR) subgroup analysis based on exercise type.


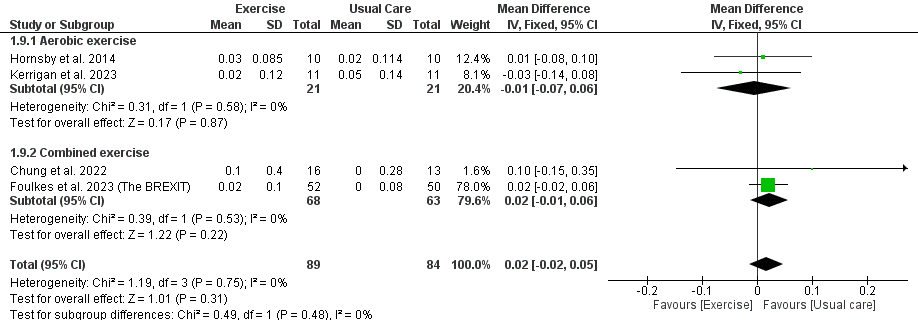


Figure S16: Respiratory exchange ratio (RER) subgroup analysis based on exercise type.


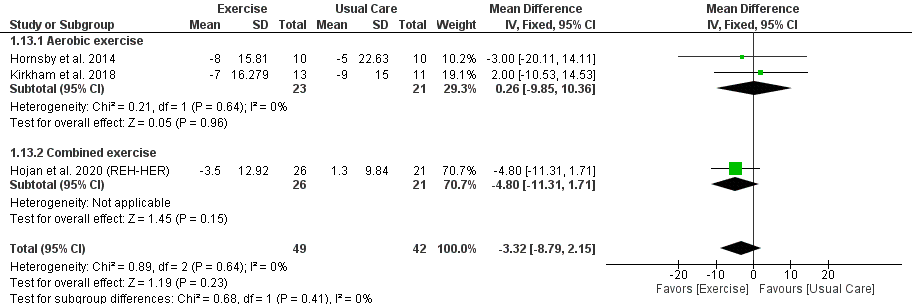


Figure S17: Resting systolic blood pressure (RSBP) subgroup analysis based on exercise type.


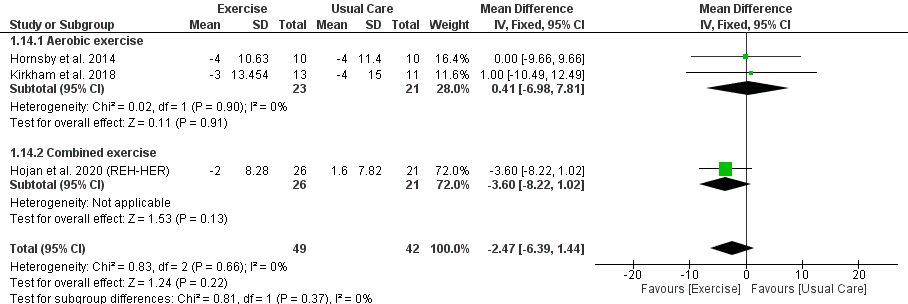


Figure S18: Resting diastolic blood pressure (RDBP) subgroup analysis based on exercise type.


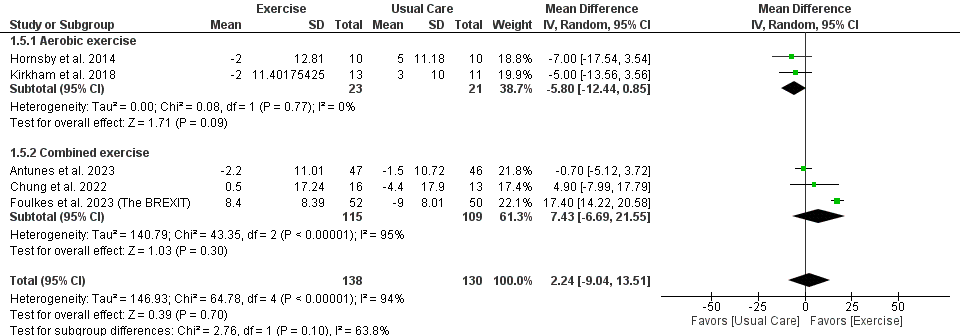


Figure S19: Stroke volume (SV) subgroup analysis based on exercise type.


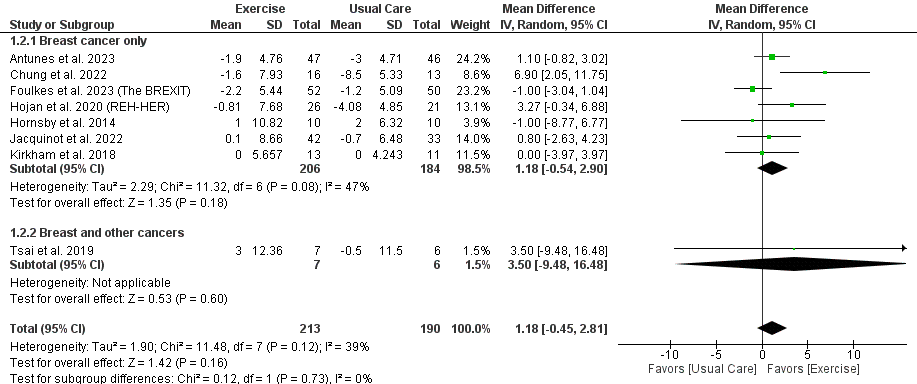


Figure S20: Left ventricular ejection fraction (LVEF) subgroup analysis based on whether the patients had breast cancer only or breast cancer plus other cancers.


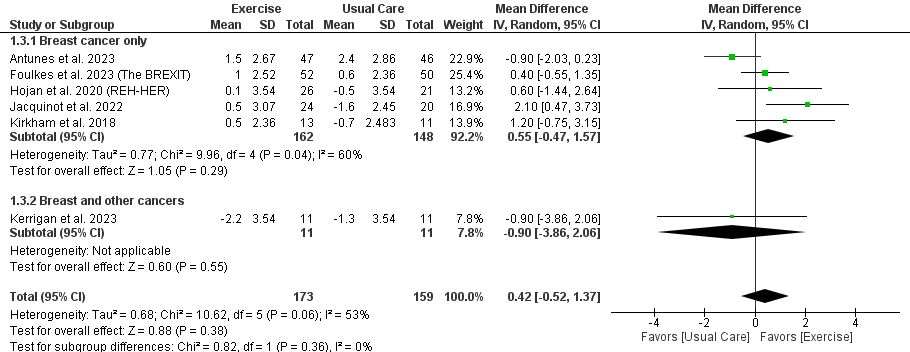


Figure S21: Global longitudinal strain (GLS) subgroup analysis based on whether the patients had breast cancer only or breast cancer plus other cancers.


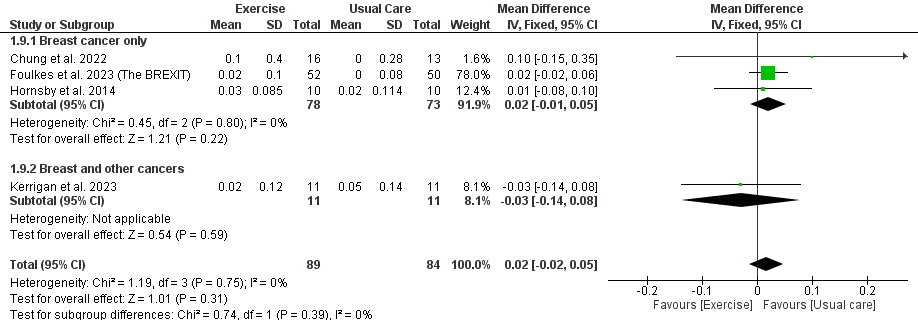


Figure S22: Respiratory exchange ratio (RER) subgroup analysis based on whether the patients had breast cancer only or breast cancer plus other cancers.


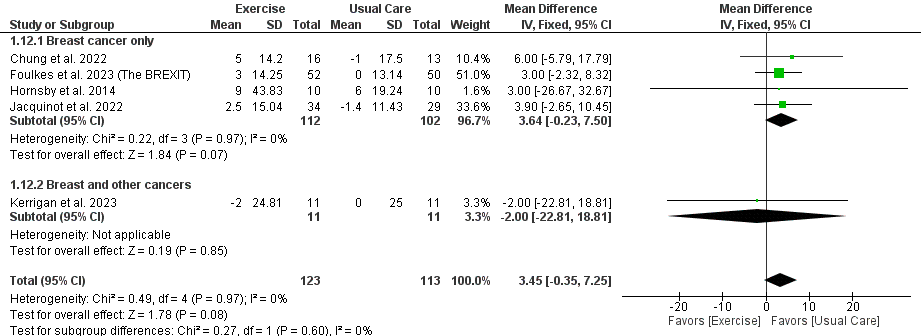


Figure S23: Peak heart rate (PHR) subgroup analysis based on whether the patients had breast cancer only or breast cancer plus other cancers.
